# The Zoom solution: Promoting effective cross-ideological communication online

**DOI:** 10.1371/journal.pone.0270355

**Published:** 2022-07-20

**Authors:** Ashley L. Binnquist, Stephanie Y. Dolbier, Macrina C. Dieffenbach, Matthew D. Lieberman

**Affiliations:** Department of Psychology, University of California, Los Angeles, Los Angeles, California, United States of America; Georgia State University, UNITED STATES

## Abstract

The rise of ideological polarization in the U.S. over the past few decades has come with an increase in hostility on both sides of the political aisle. Although communication and compromise are hallmarks of a functioning society, research has shown that people overestimate the negative affect they will experience when viewing oppositional media, and it is likely that negative forecasts lead many to avoid *cross-ideological communication* (CIC) altogether. Additionally, a growing ideological geographic divide and online extremism fueled by social media audiences make engaging in CIC more difficult than ever. Here, we demonstrate that online video-chat platforms (i.e., Zoom) can be used to promote effective CIC among ideologically polarized individuals, as well as to better study CIC in a controlled setting. Participants (*n* = 122) had a face-to-face CIC over Zoom, either privately or publicly with a silent ingroup audience present. Participant forecasts about the interaction were largely inaccurate, with the actual conversation experience found to be more positive than anticipated. Additionally, the presence of an ingroup audience was associated with increased conflict. In both conditions, participants showed preliminary signs of attitude moderation, felt more favorable toward the outgroup, and felt more informed about the issue after the CIC. These results suggest that face-to-face CIC’s are generally positive and beneficial for polarized individuals, and that greater effects may be achieved through private conversations, as opposed to more public social media-like interactions. Future researchers studying ideological conflict may find success using similar Zoom paradigms to bring together ideologically diverse individuals in controlled lab settings.

## Introduction

The meteoric rise of U.S. ideological and affective polarization [1] in the past few decades has led to open hostility across the political aisle and increasing dehumanization of those with conflicting ideologies [2]. Democracies are built on and flourish through honest discourse between those with opposing points of view [3] and decades of research indicate that intergroup contact among opposing groups decreases outgroup stereotypes [4–6]. Unfortunately, difficult ideological conversations are often avoided due to fear of hostility, conflict, and extremism from the other side [7–9]. Moreover, rural and urban areas have become less ideologically integrated, creating physical barriers for those who would seek to have conversations across the ideological divide, i.e., *cross-ideological communication* (CIC) [10]. The internet would seem to be an obvious solution to the obstacle of geographical and political boundaries. Although individuals frequently wander out of their ideological bubbles online [11], armed with the cloak of anonymity and an audience that rewards extremism and conflict, internet-based CIC often becomes a hub for hostility and dehumanization rather than a respectful exchange of ideas [12, 13]. Thus, a paradox presents itself: more dialogue between ideological opponents seems to be an effective way to combat polarization, yet the most accessible tool for scaling up dialogue (i.e., online technology) tends to exacerbate the problem.

This paradox also exists in academic settings for researchers studying ideological communication. Academic institutions are becoming increasingly homogenous in ideology [14, 15], and recruiting a wide range of opinions is notoriously difficult, let alone bringing opponents into the lab to have a heated discussion. To combat this difficulty, many researchers have opted to use imagined scenarios or forecasted experiences to study ideological communication [6]. While these methodologies are useful for identifying potential solutions for polarization, it is difficult to test their effectiveness without the ability to generate CIC in the lab. The current study seeks to address this gap in the literature by comparing participants’ forecasts of a CIC to their actual experience of one, and by examining the effects of the experience on their ideological attitudes. We demonstrate the viability of using online video-chat (i.e., Zoom) for generating and studying CIC in a controlled laboratory setting. Additionally, to test how group dynamics may hinder productive engagement and to assess the ease of experimental manipulation using online video-chat paradigms, we tested the role of an attitudinal ingroup audience as a source of conflict during face-to-face CIC.

### Negative forecasts and avoidance

It is easy to imagine the dreaded Thanksgiving dinner [16]: family members yelling about politics across the table, tensions rising, and people storming off angrily. Contrary to this perception, these conversations are relatively infrequent, as most people choose avoidance over conflict in these situations [17]. Participation in CIC can be viewed as a threat to social harmony, group ties, and reputation [18, 19]. Arguing at the Thanksgiving dinner table may cause more fracture in the family than ignoring a family member’s differing ideological beliefs. As such, people often suppress their divergent or challenging views, engaging in *self-censorship*, to maintain cohesiveness and avoid ostracism [12, 18, 20]. When it comes to strangers, people may be reluctant to have any sort of interaction, let alone a CIC. Many people report feeling uncomfortable or awkward at the thought of engaging in any kind of conversation with a stranger [21]. Despite this, when people are induced to actually have these dreaded conversations, at least in non-ideological domains, they find them to be more enjoyable than imagined [21, 22]. It is likely that similar, perhaps even greater, fears exist towards having a CIC with a stranger.

These fears may be in part due to inaccurate affective forecasts of how such interactions will go, leading people to avoid these interactions [23]. A robust body of work indicates people suffer from an “impact bias” where they overestimate both the duration and intensity of negative emotion they will experience in response to aversive events [24–26]. In the case of CIC, this bias may manifest as people form a picture of how their interaction will look by drawing upon more salient forms [27] of ideological communications that are encountered regularly, such as a vicious exchange between two users on Twitter, or the latest political tirade on the news. In using these scenarios to represent the situation, the individual may be unaware of the small percentage of people that engage in that type of antagonistic communication online [28] as well as underestimate how an asynchronous setting, compared to in-person, can have a more polarizing trajectory (expanded upon in the next section below). These easily accessible, but unlikely, examples of disagreement on the issue lead the inexperienced individual to overestimate how heated the in-person conversation is actually likely to be. Also characteristic of these tendencies is the fact that regardless of what side of the ideological divide someone is on, people seem to have almost caricatured view of their political opponents, greatly misrepresenting their beliefs and behaviors in a ‘perception gap’ suggesting a ‘false polarization’ [29–32]. In reality, ideological attitudes are not as far apart as people believe [33].

Whether it is a romantic break-up, being passed over for tenure, or watching oppositional media content, people often overestimate the degree of negative emotion in response to an aversive event [23, 25], even to the extent where they forgo potentially positive interactions in favor of solitude [23]. This strongly suggests that the actual experience of a CIC may not be as bad as people think, or even positive in nature. However, to our knowledge, no experimental research has investigated real-time, interpersonal CIC due to the difficulty of facilitating it in a controlled lab setting. The current study examines these forecasting biases in the context of face-to-face CIC’s. If similar forecasting biases are present for face-to-face CIC’s, future interventions that mitigate these biases could prove useful.

Even when psychological barriers do not prevent ideological intergroup contact, there are still growing political-geographical barriers to contend with. In the U.S., polarization along geographical lines has increased substantially over the past few decades [10]. Numerous studies have found that liberal polarization prevails in highly dense urban areas and conservative polarization prevails in rural areas [34, 35]. Existing demographics and self-selection drive ideological homogeny at the state, city, and neighborhood levels [10, 34, 35]. For those who would seek out CIC, extra effort is required in most geographic areas, potentially at the expense of alienating oneself from the community [36]. For researchers, this translates into ideologically homogenous samples on campus, making it especially difficult to study CIC.

### The internet: Echo chambers, anonymity, and the audience

In its infancy, many researchers saw the internet as an opportunity to overcome the boundaries of geography and social anxiety when it came to CIC [37, 38]. However, the internet has failed to provide the solution many thought it would, as individuals tend to inhabit online ‘echo chambers’ or ‘bubbles’, often driving people further to the extremes [39]. This is somewhat paradoxical, as recent evidence shows spending more time on social media increases exposure to diverse ideological content [40]. However, simply encountering an opponent’s ideas online is not enough to reduce hostility; if anything, merely encountering it may fuel polarization [41]. In an environment where most interactions flow one-way or are text-based, such as social media sites, communication can become extreme, incendiary, and lacking in nuance.

Notably, online CIC typically lacks the nonverbal cues, such as tone of voice and facial expression, that promote greater empathy and perspective-taking during face-to-face interactions [42–44]. Without nonverbal cues that provide feedback about an individual’s emotions and intentions, miscommunications can quickly devolve into an unproductive “Twitter war” or “Facebook fight”. On some discussion forums (e.g., Reddit), the ability to post anonymously removes social pressures for civil dialogue including accountability, possibility for retribution, and fear of isolation. Disinhibited, conversation partners are more likely to use hyperbole, dehumanization, and engage in the conversation as if it were a zero-sum game [42]. Online spaces are widely perceived as unwelcoming towards nuanced discussion [9, 45], and many individuals hide their political ideology on social media due to a lack of civility [46, 47].

Online audiences strongly influence what content social media users decide to post, comment, or respond to. Individuals online often feel like they are in a perpetual arms race to keep people’s attention, with incentives ranging from self-validation to profit. Natural human tendencies to seek out exciting new information [48] are easily co-opted by highly emotional [49] and potentially misleading information [50]. Some individuals thrive on posting novel, but inflammatory content to convey a morally righteous self-image, elicit positive feedback, or solidify ingroup status [51–53]. Although this provocative content gains traction easily, it is likely to misrepresent, dehumanize, or contain false information about an outgroup, discouraging productive CIC [49, 50, 54].

Furthermore, as online content can be seen by both attitudinal allies and opponents, users must constantly keep in mind that they are likely speaking to a mixed audience whenever they have a public interaction on social media. Past research on this “multiple audience problem” has indicated that in these kinds of situations, individuals often attempt to convey two opposing messages at the same time, one message of truth to the ingroup and another message to deceive the outgroup [55–58]. However, doing so may detract from people’s ability to engage with the actual discussion at hand–likely contributing to the unproductiveness of online CIC. Additionally, the presence of these “attitudinal ingroup” audiences may create a social pressure to espouse extreme opinions [59] to avoid “looking weak”. While social media may be the most common medium where CIC requires sending these complex messages, it is likely that any form of CIC in the presence of a mixed audience will suffer similarly. As such CIC may tend to be more productive in a private one-on-one setting than in a group.

### The Zoom solution

Video-chat software, such as Zoom, is uniquely positioned to resolve both aspects of the CIC problem, namely, the need for more conversations to combat polarization, and researchers’ difficulties in studying CIC in the lab. While the typical online interaction may be bogged down by the lack of nonverbal cues, ingroup audiences, and dehumanization, face-to-face video-chat allows for more personal conversations, allowing for online CIC more similar to in-person interactions. Additionally, the ability to connect people from across the country with just a few clicks of the mouse removes the problem of the geographical divide for citizens and researchers alike, greatly reducing the difficulty of bringing together ideological opponents. Though video communication has been around for quite some time, utilization by the general public has been slow and uneven. The need to socially distance during the COVID-19 pandemic has accelerated the adoption of Zoom to the point where it is now an eponym like Kleenex and Xerox. Many bridge-building coalitions have already begun to utilize video-chat to facilitate CIC (e.g., Living Room Conversations, Make America Dinner Again, America Talks). However, the tool has not yet gained traction among experimental researchers and thus its effectiveness as a tool has not been assessed empirically. Online tools like mTurk and Qualtrics revolutionized survey data collection; Zoom could similarly revolutionize the study of dyadic and group interactions. The familiarity and availability of video-chat means most participants will require little to no instruction on how to use the tool. Common barriers for non-college student participation in research such as travel time and location are eliminated, as individuals can participate from anywhere that has a reliable internet connection. Much like a laboratory setting, the environment on Zoom is easily controlled by sending participants to breakout rooms, changing usernames, and recording and monitoring the communication while remaining unseen. Furthermore, compared to lab-executed research with students, participants recruited online do not have to worry about the possibility of future contact with the other participants, decreasing fears of reputational damage and loss of existing relationships. In essence, Zoom chats capture the ‘sweet spot’ of allowing strangers from diverse backgrounds and locations to see one another in a maximally humanizing way in the short-term, without introducing audience effects and long-term concerns of future interactions.

### Overview of the current research

The goals of the current research were twofold. First, we were interested in characterizing the nature and effects of face-to-face CIC and how these might differ from the forecasts people make about them prior to having one. This was done by assessing the extent to which individuals had negative and inaccurate affective forecasts about a Zoom-based CIC, in addition to examining the impact these conversations had on their ideological attitudes. Second, we demonstrate how researchers may use similar Zoom-based paradigms to experimentally manipulate aspects of ideological communication in controlled settings by testing whether the presence of attitudinal ingroup audiences may induce greater levels of conflict and other negative outcomes. Simulating conditions that increase conflict like those seen in social media interactions can help inform the psychological mechanisms that influence conflict online and in-person. To do this, we recruited people with opposing viewpoints and brought them together on Zoom. All participants first had an ingroup ideological conversation alone with an attitudinal ingroup member who shared their views. After this initial conversation, participants had a CIC conversation with an attitudinal outgroup member holding the opposing view. In half of the CIC conversations, the ingroup member from the first conversation was in the Zoom call silently observing (i.e., *public* condition) and the other half had a conversation with no ingroup member watching (i.e., *private* condition). Level of conflict was assessed both by participant self-report and a set of independent coders.

## Results

### Affective forecasts

**Fig 1:**
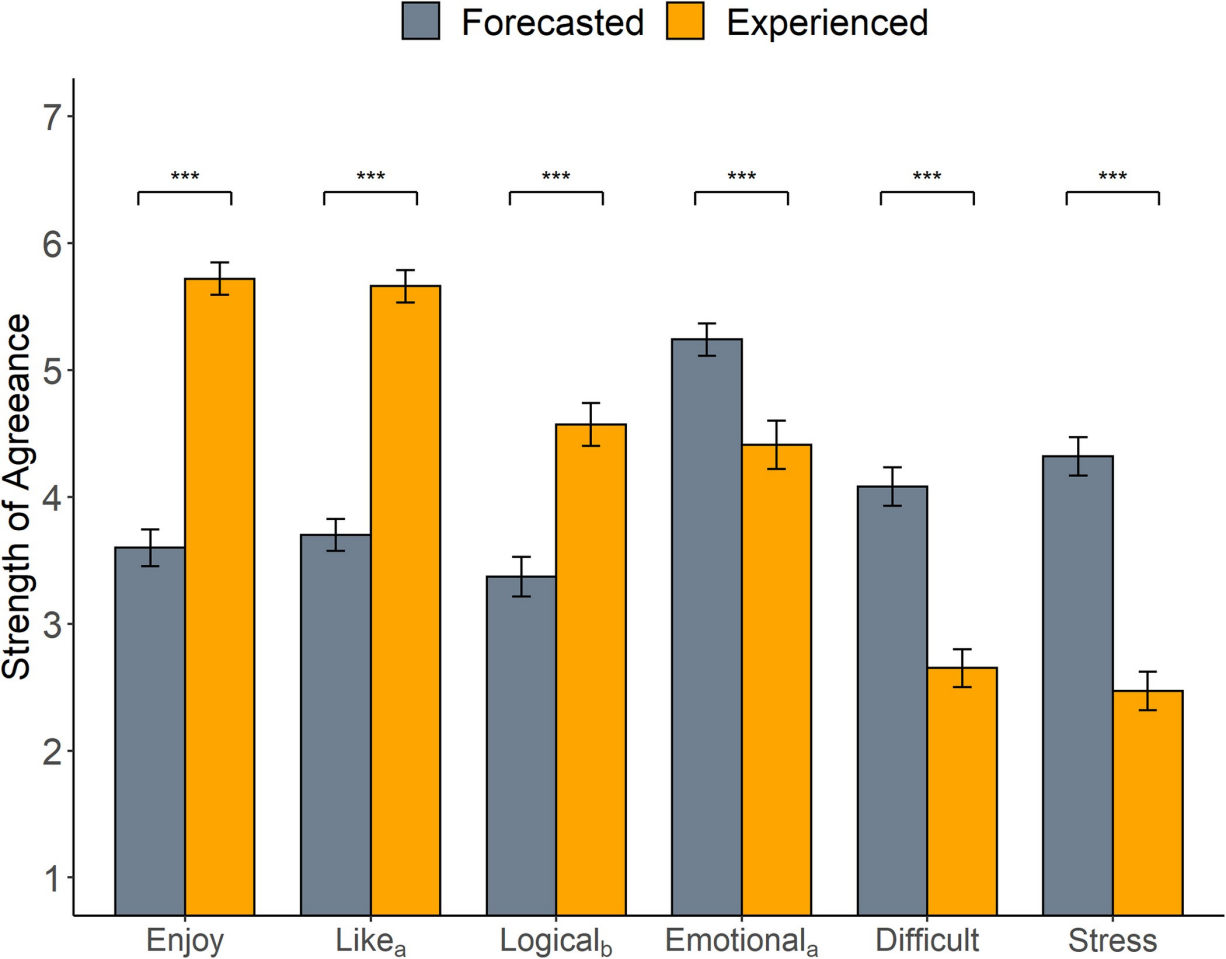
Difference in CIC forecasts vs experience. *Note*. Difference in what participants (*n* = 120) forecasted about the CIC beforehand, compared to what they actually experienced during the CIC. Variables were measured on a 1 (strongly disagree) to 7 (strongly agree) scale. For example, “I believe this interaction would be enjoyable” and “This interaction was enjoyable”. For exact wording of questions and additional statistics, see Table S2 in S1 Appendix. ***p < .001, ^a^ Sample size is *n* = 106, ^b^ Sample size *n* = 105.

Participants’ forecasts about how a conversation with an attitudinal outgroup member would go were contrasted to their actual experience of having such a conversation (see Table S2 in S1 Appendix for the full list of CIC forecasts). The following results are presented independent of either the *public* or *private* condition as no significant differences were found between conditions (for condition-based analyses of affective forecasting differences see Table S6 in S1 Appendix). On average, participants reported experiencing less time in conflict during the CIC than they forecasted (*t* = 9.85, *p* < 0.001, Cohen’s *d* = -1.19). As shown in Fig 1, participants also reported that the conversations were more enjoyable (*t* = 13.44, *p* < 0.001, Cohen’s *d* = 1.28), less stressful (*t* = 11.04, *p* < 0.001, Cohen’s *d* = -1.11) and less difficult (*t* = 7.94, *p* < 0.001, Cohen’s *d* = -0.86) than they predicted. Additionally, participants tended to like their CIC partner more than expected (*t* = 13.48, *p* < 0.001, Cohen’s *d* = 1.50), and found them to be less emotional (*t* = 4.06, *p* < 0.001, Cohen’s *d* = -0.50) and more logical (*t* = 7.05, *p* < 0.001, Cohen’s *d* = 0.72) in their arguments than anticipated. Several other measures were collected (see Table S5 in S1 Appendix), showing the same general trend of these conversations being a more positive experience than participants originally believed. The gap between expectation and experience is exhibited even by individuals who reported experiencing higher levels of conflict in their CIC, with ratings of the conversation and their partner still being more positive than anticipated (analyses detailed in Table S5 in S1 Appendix).

**Fig 2:**
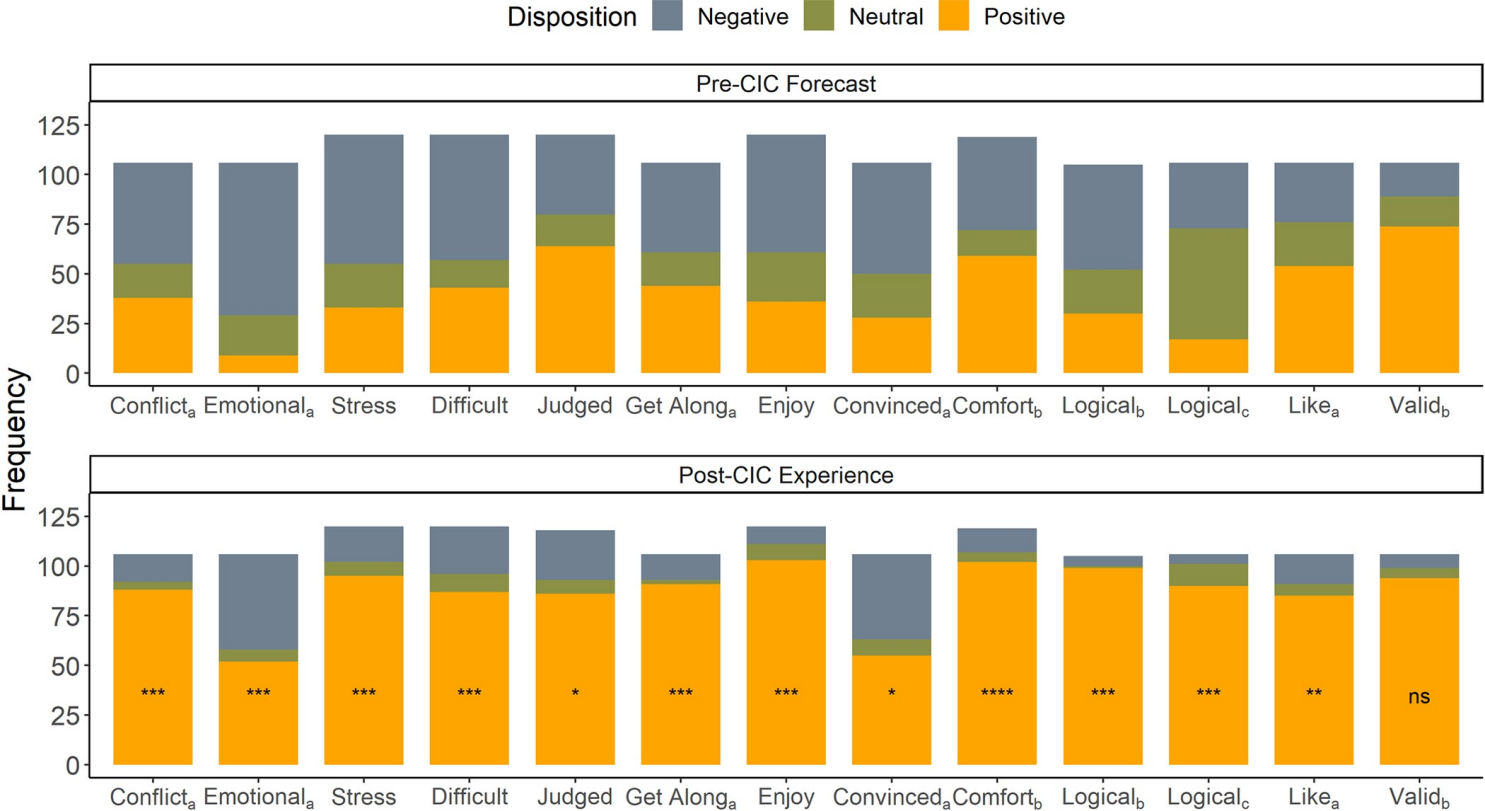
Proportion of disposition for pre-CIC and post-CIC forecasted/experienced variables. *Note*. Chi-square analysis of each variable (*n* = 120) was significant for a difference in the proportion of disposition. Looking at the graph a larger proportion of responses look to be in the positive disposition post-CIC. Pairwise analysis of the negative vs positive proportion (noted by Bonferroni adjusted p-value asterisks) and the neutral vs positive proportion confirmed the omnibus test, such that a larger portion of positive disposition compared to either negative or neutral disposition was the direction of change. *p < .05 with Bonferroni correction. **p < .01 with Bonferroni correction, ***p < .001 with Bonferroni correction, ^a^ Sample size is *n* = 106, ^b^ Sample size *n* = 105, ^c^ Sample size *n* = 119.

We next examined whether forecasts were qualitatively different from experiences such that overall disposition (i.e., the combination of valence and intensity) of the experience was mis-predicted. Unlike the vast majority of affective forecasting studies for which the valence of forecasts and experiences are the same, but intensity is mis-predicted, here participants reliably mis-predicted the disposition of the experience typically predicting they would have (or be in) a negative disposition when in fact they had (or were in) a positive disposition. A chi-square goodness of fit test was calculated comparing the proportion of negative, neutral, and positive disposition for post-CIC experience to the proportion of disposition in the pre-CIC forecast variables. Significant deviation from pre-CIC forecasts were found for all variables tests, including stress (χ^2^ = 64.40, *p* < .001), difficulty (χ^2^ = 33.46, *p* < .001), and enjoyment (χ^2^ = 77.82, *p* < .001), (for full list of chi-square statistics see Table S7 in S1 Appendix). As shown in Fig 2, the change in proportion of disposition is driven by the number of participants who forecasted a negative disposition pre-CIC but experienced a positive disposition post-CIC. Post-hoc pairwise comparisons of proportional difference were significant after Bonferroni correction for the proportion of negative to positive disposition for all but one of the variables tested, such that a larger proportion of participants were in the positive disposition post-CIC. For ten of the thirteen variables tested there was also a change in proportion of neutral and positive disposition that favored the positive disposition post-CIC (for full list of pairwise significance see Table S7 in S1 Appendix). Ten of the thirteen variables also had a complete flip of proportion from negative to positive disposition such that not only did the proportion change in favor of a positive disposition, but the majority share of the proportion changed from the negative disposition to positive disposition post-CIC.

### Issue-based changes

To examine what effect engaging in a CIC had on participants’ ideological attitudes, their responses on a series of issue-based measures were compared between pre- and post-conversation. Similar to the affective forecasting results, the following set of findings are presented independent of condition. Participants felt more informed on the issue that was discussed (*t* = 4.88, *p* < 0.001, Cohen’s *d* = 0.43), cared more about it (*t* = 3.01, *p* = 0.003, Cohen’s *d* = 0.28), and perceived it to be more important (*t* = 2.74, *p* = 0.007, Cohen’s *d* = 0.28) after having a CIC. In addition to this increase in interest towards the issue, participants became less confident from pre- to post-study that their attitude on the issue was any more correct than the opposite attitude (*t* = 3.69, *p* < 0.001, Cohen’s *d* = -0.34). Additionally, their reported attitudes on the issue became less extreme on average, moving towards the center (*t* = 2.49, *p* = 0.014, Cohen’s *d* = -0.27), coders also perceived a similar shift towards attitude moderation from pre- to post-CIC (*t* = 5.46, *p* < 0.001, Cohen’s *d* = -0.30). Participants’ feelings toward their attitudinal outgroup also improved from pre- to post-study: participants felt more favorable towards those people with the opposite opinion as them on the issue (*t* = 4.56, *p* < 0.001, Cohen’s *d* = 0.49). Full analysis can be found in the Table S9 in S1 Appendix as well as condition-based differences in Table S10 in S1 Appendix. Together these results highlight a shift towards attitude moderation after engaging in a CIC.

### Audience effects

The role of ingroup audience was analyzed with both self-reported and coder-rated conflict during the CIC as a factor of condition (i.e., *private* vs. *public*). During the CIC, participants in the *public* condition self-reported experiencing more time in conflict on average compared to participants in the *private* condition (*t* = 2.03, *p* = 0.044, Cohen’s *d* = 0.37). Corroborating the self-report data, independent coders also perceived more time in conflict on average in the *public* condition compared to the *private* condition (*t* = 2.17, *p* = 0.032, Cohen’s *d* = 0.39). Participants in the *public* condition also felt the CIC was more difficult than did participants in the *private* condition (*t* = 2.44, *p* = 0.016, Cohen’s *d* = 0.44) as did coders (*t* = 2.17, *p* = 0.032, Cohen’s *d* = 0.39). Coders also perceived more stress (*t* = 3.40, *p* < 0.001, Cohen’s *d* = 0.62), less enjoyment (*t* = 3.55, *p* < 0.001, Cohen’s *d* = -0.64), and less respect (*t* = 2.55, *p* = 0.012, Cohen’s *d* = -0.46) in the *public* condition. Additional analysis of condition differences for self-report and coders can be found in the Table S11 in S1 Appendix.

Moreover, questions only asked of coders about the CIC showed a similar trend of more negativity with coders reporting more frustration (*t* = 2.62, *p* = 0.010, Cohen’s *d* = 0.47), bad faith arguing (*t* = 2.11, *p* = 0.037, Cohen’s *d* = 0.37), and a more inflammatory tone (*t* = 2.05, *p* = 0.043, Cohen’s *d* = 0.38), in the *public* versus *private* condition. There was also more private disagreement (*t* = 2.22, *p* = 0.029, Cohen’s *d* = 0.40), in the *public* versus *private* condition based on coder ratings, but not outward disagreement (*t* = 1.48, *p* = 0.140). Similarly, coder ratings of the CIC as a dyad found more hidden conflict (*t* = 2.56, *p* = 0.013, Cohen’s *d* = 0.64) in the *public* versus *private* condition, but no difference in overt conflict (*t* = 0.98, *p* = 0.331), or overall conflict (*t* = 1.79, *p* = 0.079), For additional analysis of coder only ratings see Tables S12, S13 in S1 Appendix.

**Fig 3:**
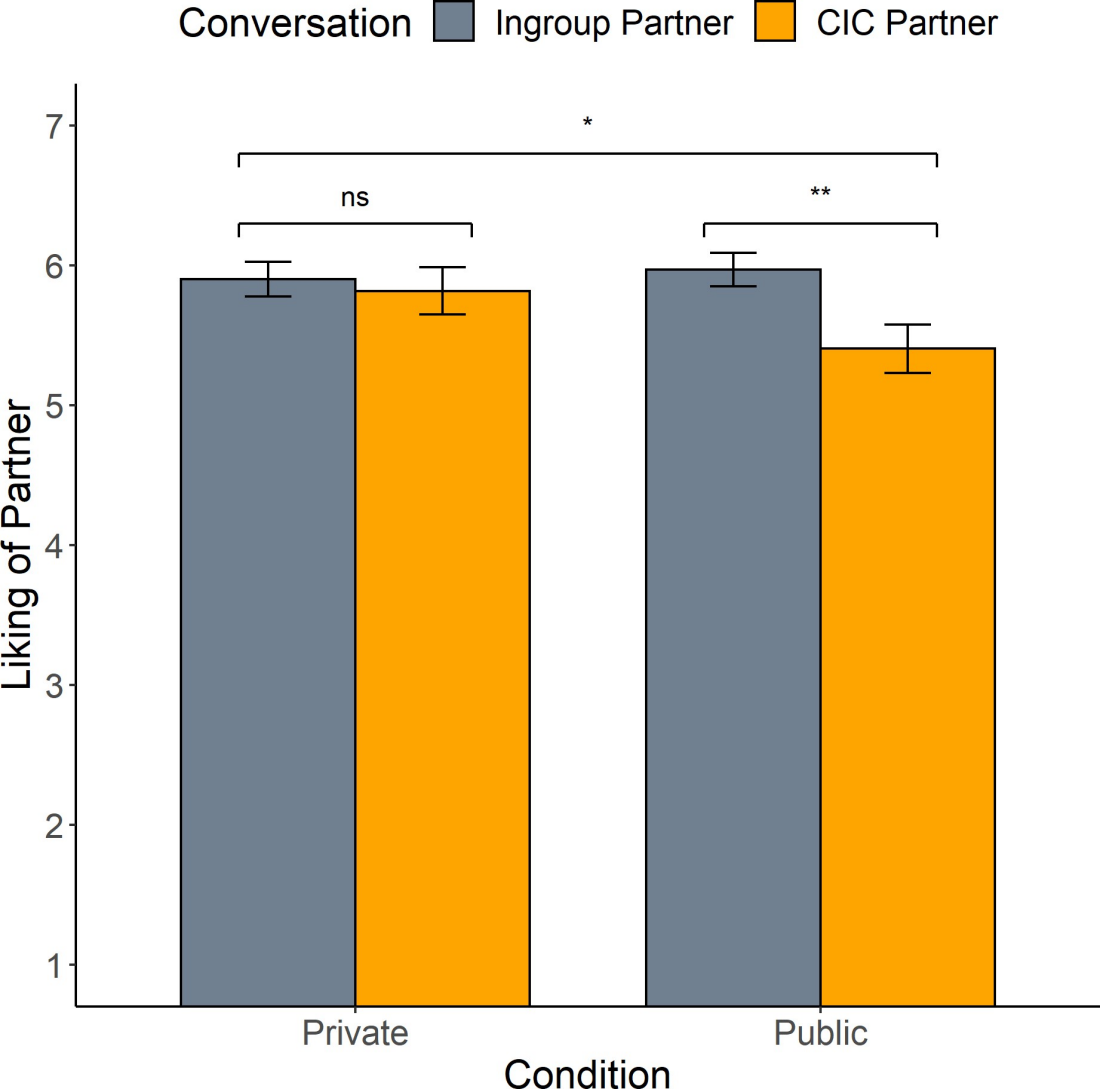
Strength of conversation partner liking: Condition by partner. *Note*. Shown here is liking of conversation partner, reported on a scale of 1 (strongly disagree) to 7 (strongly agree). Driving the interaction of condition by partner (*n* = 122) is the difference in liking of conversation partner for the *public* condition, such that participants liked their attitudinal ingroup member from the initial conversation more than their CIC partner. * p < .05 for the main effect of partner (i.e., ingroup vs CIC-partner). **p < .01 for the simple comparison of ingroup partner vs CIC-partner in the *public* condition.

As shown in Fig 3, when participants spoke *privately* in their CIC, they liked their outgroup partner from the second conversation just as much as they liked their attitudinal ingroup member from the initial conversation (*t* = 0.50, *p* = 0.62, Cohen’s *d* = -0.07). However, when the ingroup member was silently observing the second conversation (i.e., *public*), this led to significant drop in liking for the CIC outgroup partner (*t* = 3.26, *p* = 0.002, Cohen’s *d* = -0.41). The full interaction between condition and conversation partner was also significant (*F* = 4.04, *p* = 0.047; full analysis in Table S14 in S1 Appendix). Though we did not have coders rate the initial conversation with the attitudinal ingroup member, the coders did perceive a significant difference for partner liking in the CIC (*t* = 3.49, *p* < 0.001, Cohen’s *d* = -0.63), such that in the *public* condition coders perceived participants liking the conversation partner less than those in the *private* condition.

### Coder time course analysis

**Fig 4:**
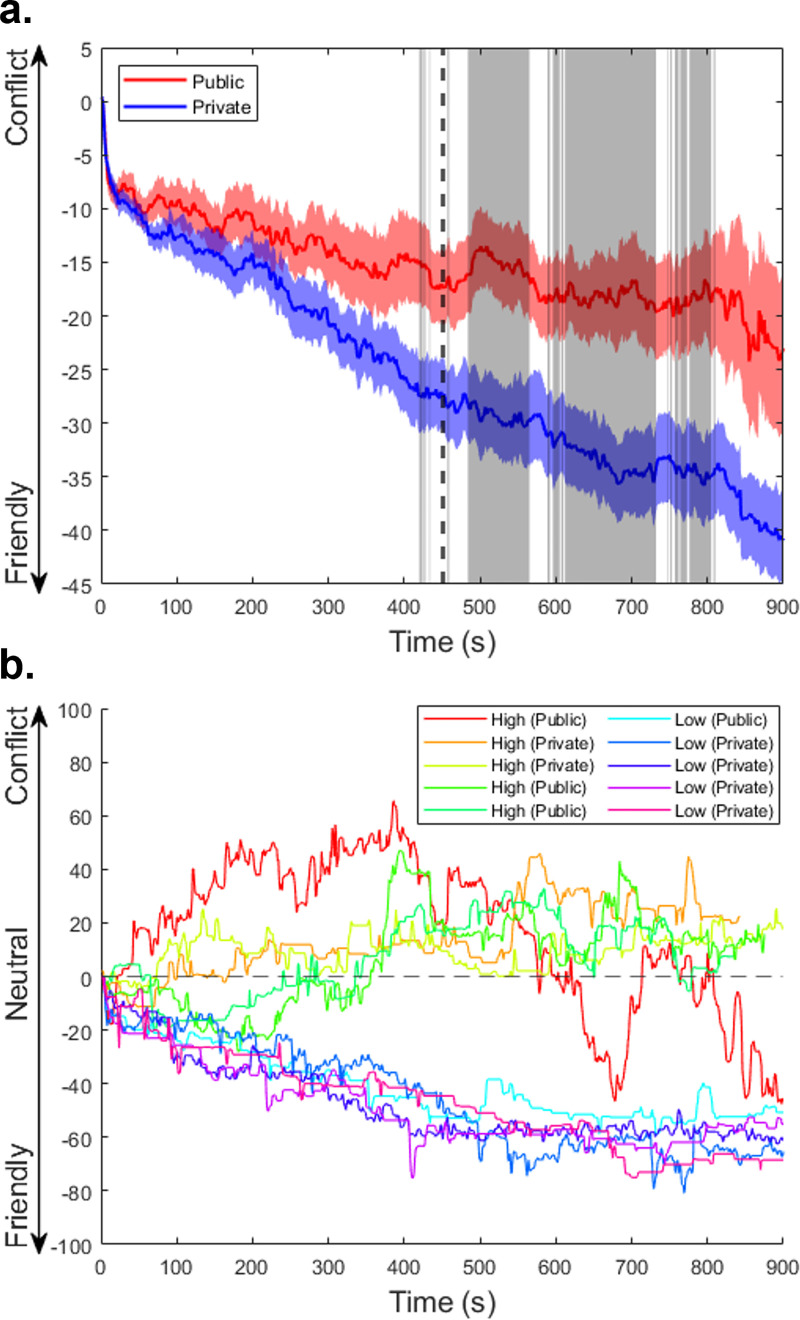
Coder-rated conflict time courses of conversations. *Note*. **a.** Mean time courses of coder-rated conflict are shown for both the *public* and *private* conditions. The gray shaded regions show timepoints where the difference between conditions is significant. These regions are mostly confined to the latter half of conversations, with the first major region beginning at 484 seconds. Colored shaded areas represent +- 1 SE across conversations. **b.** Top 5 and bottom 5 conversations’ time courses as ranked by coder-rated conflict. Both *public* and *private* conditions are represented within each grouping, showing that while *public* conversations may have more conflict on average, conflict within any individual conversation is still highly variable.

Coders additionally made continuous ratings of the amount of conflict over the time course of each conversation. Fig 4A shows the average time courses by condition. An independent samples t-test was run at each timepoint, using the two-stage Benjamini, Krieger, & Yekutieli FDR correction [60], to quantify differences between the average time course of each condition. Conversations in the *public* condition were rated as having more conflict than conversations in the *private* condition, with these differences appearing in mostly in the second half of the conversations (the first large chunk of significance appearing at 484 seconds; *q* < 0.05, *p* = 0.018). Averaging over time to produce an aggregate measure, these continuous ratings show the same pattern of *public* conversations being rated as higher in conflict than *private* conversations (*t* = 2.78, *p* = 0.007, Cohen’s *d* = -0.71) that was found in the overall rater ratings of conflict reported in the previous section.

Following up on the apparent trend seen in the time course data, a post-hoc analysis of the differences between time courses across conditions showed that while there was no significant difference in coder-rated conflict between *public* and *private* conversations in the first 450 seconds of these conversations (*t* = 1.76, *p* = 0.084, Cohen’s *d* = -0.45), *public* conversations were rated as having significantly more conflict than *private* conversations in the latter 450 seconds (*t* = 2.73, *p* < 0.008, Cohen’s *d* = -0.70).

Looking at Fig 4A, it is clear that on average, conversations began somewhat neutrally and improved from there with average levels never rising above 0 at any timepoint. However, individual conversations were much more variable. The top five and bottom five conversation time courses as ranked by coder-rated conflict are shown in Fig 4B. Those conversations with the highest coder-rated conflict often spent a significant portion of the conversation above 0.

## Discussion

Overall, the results of this study are promising for the potential of video-chat paradigms to facilitate effective CIC, both for participants and for researchers. Perhaps the most consequential results involved affective forecasting, as participants largely overestimated the negative affect that they would feel during a CIC. Participants found that the CIC experience was more enjoyable and less stressful than they had anticipated, with essentially only half as much conflict as predicted. Participants also found that the other side tended to be more logical and less emotional than expected. Furthermore, participants found that they liked their conversation partners as individuals, despite having negative opinions about them prior to the experiment.

These inaccurate forecasts demonstrate the reality that many people have misconceptions about their ideological opponents, highlighting the so-called ‘perception gap’. It may be that these initially negative perceptions contribute to avoidance, creating barriers to engaging in CIC [20–23], and ultimately preventing these misperceptions from ever being corrected. Although future studies are needed to investigate the role these misperceptions play in discouraging CIC, one participant’s comment after opting out of the study prior to the conversation lends support for this possibility:

I am not sure that I can trust [the person I would be talking to]… I don’t know if they will try to do something psychotic if they don’t like what I said. …recently people are not acting rationally and are attacking people just because they don’t completely agree with them.

In contrast, many participants who actually completed the CIC wrote about how misinformed their initial assumptions about a CIC partner were. One participant in particular summed up what many said throughout the comments:

The person from the second conversation was not who I was expecting. I pictured someone more stereotypically conservative, bigoted, and likely to recite propagandized arguments founded in emotion/fear/religion. Admittedly, I prejudged the participant before I met them. I was pleasantly surprised to meet someone much different, whose reasons for opposition were intelligent and founded in logic. The conversation did not convince me to change my opinion on the issue, but it did open me up to another perspective.

This comment portrays the majority of participants whose forecasts like stress, difficulty, and enjoyment fell within the negative disposition range but flipped post-CIC into the positive disposition range (see Table S16 in S1 Appendix for full list of comments). Future work on how to encourage CICs may benefit from seeking to reduce people’s initially negative perceptions of such interactions, perhaps by preemptively informing them of the fact that most people end up enjoying the experience. Indeed, one of the best ways to encourage more CICs may be to raise public awareness about the misperceptions that we have about them.

While some research has shown that viewing oppositional media can increase people’s polarization [41], it appears that face-to-face conversations may have an advantage when it comes to engaging with the other side. An especially heartening result for researchers seeking to reduce ideological polarization is that in many cases, attitude moderation and more favorable dispositions towards the attitudinal outgroup were observed after engaging in CIC. Neither of these results were specific to either the *public* or *private* condition, so it seems that just having a conversation with someone with different views may have helped inject nuance into people’s ideological views. Furthermore, like previous research that has looked at affect post-CIC on Reddit [61], participants felt more informed about the issue and more positive about similar types of communication in the future.

Similar to previous research showing that online audiences can lead to more antagonistic behaviors [51], the current research found that having a silent ingroup audience present in a face-to-face CIC led to more conflict. Two possible mediating factors may help explain why the presence of an ingroup audience leads to more conflict. The first may be that in the presence of an ingroup audience participants felt more emboldened to speak up about their beliefs which generated more conflict with their cross-ideological partner. One participant’s comment exemplifies this, “Having the other two folks watching was really nerve-wracking, but occasionally I’d see my fellow [partner] nod [along] and it made me feel reassured.” The second possible mediating factor for more conflict in the *public* condition is that participants felt more pressure to defend their attitude, or wanted to avoid looking disingenuous, when someone from their ingroup, with whom they had already spoken, was present. This account is consistent with Heider’s balance theory [62] and would explain why participants in the *public* condition felt the CIC was more difficult than those in the *private* condition. The idea that the need to defend an attitude when an ingroup is present is supported by the coder’s perception that participants engaged in more unproductive types of conflict like frustration and bad faith arguing. However, no difference in the ability to speak openly about the topic was found between conditions, based on self-report and coder ratings. This may explain why coders also perceived less listening in the *public* condition as participants may have been more focused on portraying a positive image to their ingroup, by vigorously defending their shared attitude, than engaging in a productive communication with their opposition partner. These findings also give insight into why cross-ideological interactions on social media are generally antagonistic. Individuals posting a comment, or a tweet know that it will be seen by a wide array of people containing both ideological allies and opponents. Rather than simply posting their honest opinion or thoughts about something, they must navigate a complex landscape of engaging with the outgroup while signaling loyalty with their ingroup. This may turn what might have been a productive dialogue into simply trying to find the best “dunk” on one’s opponents. Future research should look into this possible contributor to the toxicity of online CIC–it may be that hostility can be reduced simply by moving conversations out of the public sphere (e.g., “Let’s continue this conversation in private”).

Coder-rated conflict revealed that the difference in conflict between conversations in the *public* and *private* conditions was mostly driven by the second half of conversations. Notably, as seen in the average time courses, *public* and *private* conversations both seem to get friendlier over time on average; however, having an audience appears to reduce the rate at which this occurs. This may be because the presence of ingroup audience makes it more difficult to open up to one’s supposed opponent, inhibiting potential coalition-building that would otherwise result in a friendlier conversation. One important qualification for this time course data is that conversations tended to open with both participants taking turns describing their own attitudes, generally taking up the first few minutes. This may be why the difference between *private* and *public* conditions only becomes apparent later into the conversation, as participants could only begin to engage with one another after first establishing their positions. Further behavioral coding and language analyses ought to be done to fully examine the temporal dynamics of these CIC, thus the speculations about the meaning of the time course data presented here is only preliminary.

Overall, the success of the current paradigm paves the way for Zoom-based paradigms to become a staple for researchers studying CIC. The face-to-face nature of conversations avoids the pitfalls of traditional internet-based conversations [5, 43], but the virtual aspect may make participants feel more comfortable due to greater anonymity than would be possible in-person, preventing conversations from feeling artificial or forced. Of course, future work is needed to investigate the full mechanisms behind how Zoom CIC differs from online or in-person CIC. Nonetheless, the current study has found great success both using Zoom to facilitate effective CIC, and to ask experimental questions about the factors that influence it.

Of particular note is how using Zoom in conjunction with mTurk mitigates the largest concern that traditionally plagues online-only subject pools [63]. The data quality of online samples is often called into question due to high degrees of inattentive and fraudulent response, since performing asynchronous tasks through a computer screen is generally low effort and easy to fake. In contrast, showing up to a face-to-face Zoom interaction at a pre-scheduled time requires much more commitment and engagement on participants’ parts, making participants who do show up more similar to those who might be recruited for an in-person laboratory study as opposed to those clicking through a traditional mTurk study. This allows researchers to reap the benefits of online subject pools while avoiding the largest downsides.

Like any research that requires voluntary participation, the current study may be subject to the problem of self-selection. Many participants chose not to continue once they found out the study entailed talking to another participant of the opposite attitude face-to-face. In fact, although we received 4231 responses on mTurk to the initial prescreener, only a total of 2169 mTurkers reported strong enough attitudes to qualify for the experiment. Of these, only 577 indicated their willingness to participate, only 247 responded to initial attempts for scheduling, and finally only 122 showed up for the Zoom conversations and completed the study. As such, the participants that did complete the study may only represent a small subset of individuals who were brave enough to take part in a face-to-face CIC, or who just enjoy a lively debate. We suspect that since even the people most willing to have CIC held such inaccurate forecasts of how the interaction would actually go, it is possible that people unwilling to have CIC may hold the same, or even greater misperceptions–driving their avoidance.

Future research would benefit from examining exactly how these self-selected participants are similar and different to the larger population of polarized individuals, perhaps providing insight into how participation in CIC might be encouraged. With that being said, we cannot differentiate the participants who dropped out because they were fearful of the CIC or because, as many researchers have become aware of in recent years [63], they were individuals who lied about their true identity through mTurk. There were many instances where a series of screeners from the exact same geographical location were submitted within minutes of each other with only one question changed each time until that pre-screener was deemed eligible for the study. The questions changed were often the English fluency, the age limit, or the strength of willingness to defend their attitude, all qualifications used to determine eligibility for the study. Additionally, many eligible participants provide fraudulent emails that bounced back when used for scheduling, so it is likely that the true number of individuals who opted not to participate is smaller than these numbers suggest.

A related limitation is that of selection bias. In order to achieve the greatest likelihood of generating CIC in the lab, we recruited participants with extreme attitudes on various issues, and excluded those with moderate attitudes. Although we reassessed participants’ attitudes again prior to CIC, there is still the possibility of the observed attitude change being partially due to regression to the mean or ceiling effects, as a majority of participants’ initial attitudes were at the extreme ends of our scale by design. Additionally, much research into persuasion and attitude change shows that the observed change in some experimental designs are often only short-term, with attitudes reverting back in the long term [64–66]. Similarly, although participants displayed a moderation of attitudes in the short term, the long-term effects of CIC intervention are unknown. As such, the observed attitude moderation effects, while promising, should be seen as tentative until more research is done. Fortunately, even if CIC is only able to produce short-term effects, we might envision a future where a culture of cross-ideological discourse is encouraged, and CIC becomes sufficiently ubiquitous that even its short-term effects can produce a lasting change on our political climate.

Another limitation of the study is the fact that all participants had a conversation with an ingroup member. This conversation was used to create a sense of ingroup for the *public* condition and was included in the *private* condition for control purposes. The conversation with an ingroup member always came first in the current study, so it may have acted as a form of practice, allowing people to revisit their beliefs before talking to an opponent. This could have prepared people to talk about their attitudes in the CIC, changing how it might have gone if this first conversation were not present. For instance, a participant might feel emboldened to defend their opinion by the first conversation, leading to more confrontation in the CIC. Alternatively, having a positive interaction with an ingroup member first may have made people more agreeable when talking to their opponent afterwards. Pilot work in our lab without the first conversation tended to produce CIC in which both participants acted as if their own position was much more in the ideological middle than it truly was. This yielded pleasant, but artificial conversations in which little meaningful dialogue or attitude change occurred. Future work is needed to understand how a pre-cursor conversation, or lack thereof, can affect people’s mood or ability to speak about an attitude during a CIC.

It is important to note that as the presence of CIC was not randomized, the study design does not allow us to separate the effect of simply having any sort of ideological discussion from that of a cross-ideological discussion specifically. It may be that simply discussing politics with a stranger may lead to more nuanced views, regardless of whether they agree or disagree. Alternatively, having any sort of conversation with an ideological opponent, whether it is about politics or something completely neutral, could lead to more positive views towards them. More research is needed to compare different kinds of interactions between polarized individuals.

## Conclusion

Rather than treat online ideological interactions as a lost cause, a better solution may be to embrace ever-improving technology and find new ways to have positive interactions. With the increased adoption of online tools such as Zoom, it is possible to break people out of their ideological bubbles to foster more productive CICs. By recruiting individuals from across the United States we were able to avoid the geographical divide, humanize ideological opponents to each other, and generate effective CIC in the lab. We hope that by highlighting the inaccuracy of negative forecasts surrounding CIC we might better foster more engagement and break down the misconceptions that pervade discussions of ideology. Similar Zoom paradigms may be a promising tool for researchers looking to develop interventions for polarization. Combatting polarization requires specific goals at every level in society. At the interpersonal level, increasing participation in CIC may be one of our most effective tools to mitigate antipathy directed at outgroups and combat the growing divide. In a moment in history when many people feel like the only sensible thing to do is to ‘tune out’ the other side, being willing to ‘log in’ and get face-to-face may be our best hope to really understand each other.

## Materials and methods

### Participants and recruitment

The study was approved by the institutional review board of the University of California, Los Angeles, including both forms of consent taken before each survey and before the conversation portion of the study. Due to the online nature of the study a consent form was presented before the pre-screener, pre-study survey, and Zoom conversations. The first form of consent was before taking the pre-screener and pre-study participants were told that by clicking the next button after the consent form they agreed that they had read the consent, their participation was voluntary, and that they were over the age of 18 years old. The second form was verbal consent given before the start of the conversation portion on Zoom after they had been given an electronic copy of the consent form. Additionally, all data was analyzed anonymously. Participants were U. S. residents that consisted of an ethnically diverse sample (for more detailed demographics see Table S1 in S1 Appendix). Primary analysis included a total of 122 participants, 59 male, 59 female, 4 non-binary aged 18–40 (*M_age_* = 30.36, *SD* = 5.65). Pre-study affective forecasts were not collected for 16 subjects due to an administrative error. Thus, affective forecasting analyses are noted where the sample size is smaller than 120 (most commonly *n* = 106) due to unanswered questions. Despite this, demographics for the sample changed very little (see S1 Appendix, Table 1).

**Table 1:** Topics discussed during CIC for the experiment.

Topics of conversations
1. The father of an unintended pregnancy should have the right to be involved in the decision-making process for an abortion. **(C)** 2. We shouldn’t force people into the categories of male or female, gender is a spectrum. **(L)**
3. Cities should defund the police to combat systemic discrimination. **(L)**
4. Colleges should use affirmative action policies, such as considering an applicant’s ethnicity, income level, etc., when deciding admissions. **(L)**
5. Private businesses should have the right to refuse service on the basis of religious exemptions. **(C)**

*Note*. To avoid ideological bias when participants read the issue statements three of the issues were phrased in the liberal **(L)** direction and two of the issues were phrased in the conservative **(C)** direction. Responses to issues 1 and 5 were later reverse coded to lean liberal for analysis.

Candidates for the study were recruited from Amazon’s mechanical Turk platform and took a brief attitudinal pre-screener based on five issues (see Table 1) to determine eligibility. Issue-based attitudes were measured on a 7-point Likert scale (1 = strongly disagree to 7 = strongly agree) and participants were considered eligible if their attitudes on at least one of the five issues fell between 1–2 (strongly agree/agree) or 6–7 (strongly disagree/disagree). Initial reports of attitudes were only used to determine eligibility. Attitudes were reassessed in a pre-study survey approximately 24-hours prior to the Zoom conversations, and again in the final post-study survey–these were the attitudes used for all analyses. For a breakdown of participant’s attitudinal ideology see Table 2. Once eligible, if participants chose to participate the study consisted of three phases: pre-study survey, Zoom conversations, and post-study survey. In the Zoom conversation portion participants first had a conversation with an ideological ingroup member (ingroup conversation) followed by a conversation with an ideological opponent (CIC).

**Table 2:** Attitudinal distribution of participants.

	Screener	Pre-study
Attitude towards Issue		
Strongly Conservative	42 (34.43%)	32 (26.23%)
Conservative	19 (15.57%)	24 (19.67%)
Slightly Conservative	-	4 (3.28%)
Neutral	-	2 (1.64%)
Slightly Liberal	-	10 (8.20%)
Liberal	28 (22.95%)	20 (16.39%)
Strongly Liberal	33 (27.05%)	30 (24.59%)

*Note*. Although participants political ideology strongly correlated with attitudinal ideology, *r* (120) = .67, *p* < .001, issue-based attitude better represents the ideological distribution of the study. Participants reported their attitudes during eligibility screening, and again 24 hours prior to the conversation portion of the study. The distribution of liberal-leaning and conservative-leaning attitudes represented in the CICs is shown.

To avoid unreliable and fraudulent responses on mTurk, strict measures were taken. Respondents on mTurk were limited to taking the pre-screener once. Additionally, on Qualtrics potential participants were restricted from multiple submissions, had to respond to an attention check, and a reliable email was gathered from eligible participants for scheduling. Furthermore, participants had to provide their availability, respond to emails for scheduling, and show up at the correct time for the scheduled Zoom session. Once participants arrived for the Zoom session if they were not who they reported to be (e.g., not fluent in English) they were told the session had been cancelled and their data was not used for analysis. This degree of intensive recruitment and communication between the potential participants and researchers did eliminate a substantial amount of potential mTurk participants who took the pre-screener. However, we do not consider this to be an attrition problem so much as a problem with unreliable and possibly fraudulent mTurkers. The most substantial degree of participant loss was from pre-screener to the attempt at scheduling. As such the screening process only benefited the study by selecting for participants that were reliable and honest about who they said they were, both commonly known problems related to the platform’s participant pool.

### Materials and Zoom procedure

The pre-study survey consisted of issue-based measures, conversation-based forecasting measures, and related demographic measures. Issue based attitude was re-assessed on the same scale as the brief pre-screener survey. Other issue-based measures included how much they cared, felt informed, and felt the issue was of importance (1 = not at all to 4 = extremely) as well as how correct they felt their attitude was in comparison to the opposite attitude. Additionally, participants were asked what attitudes other people could hold on the issue that they would find acceptable (i.e., their latitudes of acceptance). However, participants largely reported finding these questions confusing and did not understand the sliders used for them, and as such, we did not include these “latitude of acceptance” measures in analyses.

To assess feelings toward counter-attitudinal individuals we asked participants to rate how favorably (1 = extremely unfavorable to 7 = extremely favorable) they felt towards those who strongly disagree with their attitude on the issue. A series of affective and behavioral forecasts relating to the forthcoming CIC were used in the pre-study survey. Participants were told, “…you may have a conversation with someone who has the opposite opinion as you on the following issue,” and then asked to forecast how the conversation experience would be (i.e., enjoyable, stressful, difficult), what they thought the other person would be like (i.e., likable), what the other person’s arguments may be like (i.e., emotional, logical) and what percentage of the time they predicted they would be in conflict with their CIC partner. After completion of the conversation phase all issue-based measures were re-assessed on the post-study survey as well as the past tense versions of the pre-study survey forecasts, modified to ask how the actual conversation experience was, for both the CIC and ingroup conversations. For example, the pre-study survey question “I feel this interaction would be very stressful,” was changed to, “I felt this interaction was very stressful” for the post-study survey (see S1 Appendix for the full survey materials).

Before the start of the conversations, participants were held in a Zoom waiting room and let in one by one. A total of four participants were scheduled for each session. In the event that only two participants with opposing views could be scheduled at the same time, trained confederates were used in place of the ingroup conversation partners (for details on the use of confederates see S1 Appendix). To protect anonymity participant usernames were changed (i.e., Pro/Con 1 or Pro/Con 2) and were then sent to a breakout room to wait, alone, while the rest of the participants arrived. Regardless of condition, all participants first had a one-on-one conversation with a participant (or confederate) that held the same attitude, which we refer to as the ingroup conversation. Once moved to their respective ingroup breakout rooms, participants were told they held similar attitudes on the issue. Participants had a conversation about the topic for ten minutes. The structure of their conversation was designed to be open with nothing off limits, but participants were told to stay on topic. After the ingroup conversation participants were moved to either the main chat room or a new breakout room to have a CIC.

**Fig 5:**
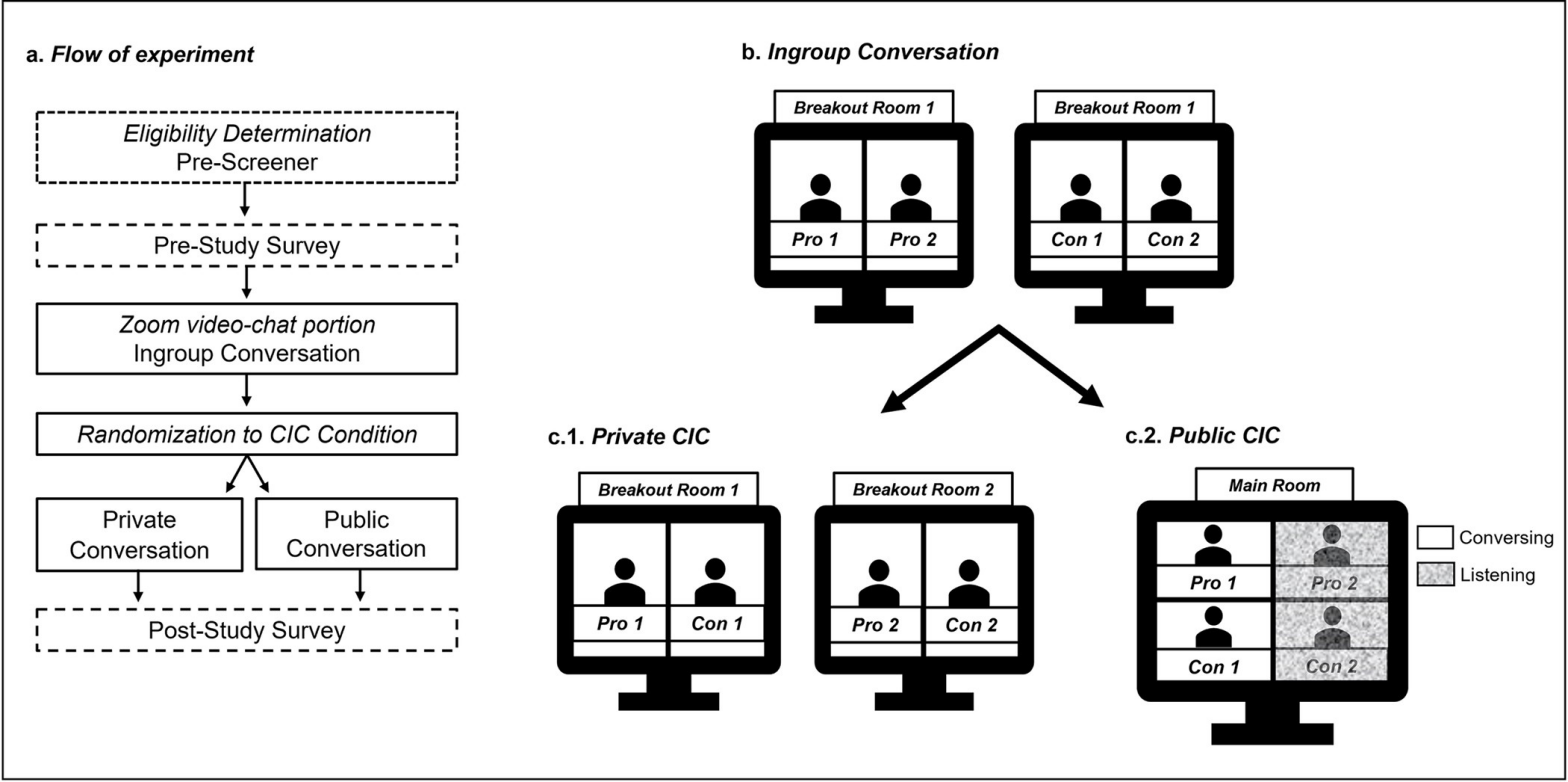
Flow of the experimental Zoom paradigm. *Note*. **a.** Flow of the study **b.** the ingroup conversation consisted of two simultaneous 10-minute conversations with two participants of the same attitude on the issue **c.** the CICs consisted of two 15-minute conversations with a participant of the opposing attitude on the issue **(c.1)** the *public* condition included all four of the participants from the ingroup conversation, with pro 2 and con 2 silently listening while pro 1 and con 1 discussed the issue, after which pro 2 and con 2 had a 15-minute CIC while pro 1 and con 1 silently listened **(c.2)** in the *private* condition two simultaneous CICs occurred in separate breakout rooms with one participant from each side of the issue.

If randomly assigned to the *public* condition, both ingroup conversation pairs (Pro and Con) were brought together in the main chat room (see Fig 5). Before the start of the conversation participants were told that the new pair of participants held the opposing attitude about the issue and that only one person from each side would take part in the second conversation first (i.e., Pro 1 and Con 1) while the other two (i.e., Pro 2 and Con 2) would be muted. The muted participants were told to keep their webcams on, pay close attention to the conversation, but to not interrupt the conversation. The CIC included the same instructions as the ingroup conversation, but fifteen minutes were given instead of ten. After fifteen minutes Pro 1 and Con 1 were stopped and told they would then listen as Pro 2 and Con 2 had a conversation about the issue. Only the first CIC (between Pro 1 and Con 1) from the *public* condition was used for analyses since the *private* condition only had one CIC. In the *private* condition, participants were separated and sent to a new breakout room with an opposing attitude participant for the CIC. For example, Pro 1 and Con 1 would be in breakout room 1, and Pro 2 and Con 2 would be in breakout room 2 (see Fig 5). Instructions for the conversation were the same as the *public* condition, sans muted participant instructions. For both the *public* condition and *private* condition, after the last conversation had ended, each participant was given a link to and completed the post-study survey.

### Coder materials and procedure

To independently assess the degree of conflict during the CICs a set of five independent coders were tasked with watching each CIC Zoom recording. Coders were blind to the hypotheses regarding the public/private conditions and were simply told that some conversations would have two participants while others would have four. Data was sampled at 20hz with a bin size of one second using the CARMA software [67]. To capture change in conversation dynamics, coders rated each conversation on a scale of -100 (friendliest conversation) to 100 (heated conflict). Coders were instructed to move the scale whenever they believed there was a change in the conversation dynamic between the conversation partners (for more detail on confederate training see S1 Appendix). For all analyses related to the CARMA ratings, we average the five coder’s ratings into a single mean time course for each conversation.

Immediately after rating a CIC video with CARMA coders were instructed to complete a post-video survey that had them assess each participant independently. Post-video survey questions were adapted from the self-report survey which included the issue-based questions and the conversation-based forecasting questions, for example “I felt this interaction was very stressful” was changed to, “The interaction seemed very stressful for them.”

### Statistical methods

Statistical analyses were conducted using Matlab R2020a and R version 3.6.3. Affective forecasting and issue-based attitude results were examined using 2-sided dependent samples t-tests. To determine the proportional change of disposition pre-CIC to post-CIC for variables that were forecasted, and experienced, responses were grouped into one of three categories: negative, neutral, or positive disposition. Categorization was a two-step process, the first step was to determine valence of the variable, that is if the question had been asked in the negative or positive. For example, “I feel this interaction was very stressful” was considered a negative valence variable. The second step was then to categorize the response of the participant as either high, low, or medium intensity dependent on the variable’s valence. For example, if a participant’s response was “disagree” to the stress question than they were categorized as having a positive disposition whereas a participant who responded “agree” to the stress question was categorized as having a negative disposition, and a participant who responded “neither agree nor disagree” was categorized as a neutral disposition. Change of disposition from pre-CIC to post-CIC for forecasted variables were tested using a chi-square goodness of fit test, with Bonferroni corrected pairwise comparisons for post-hoc analysis. Audience effects were examined using 2-sided independent samples t-tests, and the analysis shown in Fig 2 consists of a 2-way mixed ANOVA. All tests used an alpha level of 0.05. Due to the exploratory nature of this research, we did not conduct a power analysis in advance, instead choosing to aim for a sample size similar to that of other conversation-based studies [21].

## Supporting information

S1 Appendix Supplementary information including Tables S1-S16, text description of top quartile affective analysis, text description of scheduling and training, recruitment materials, and survey materials. (DOCX)
