# Supplementary material for: The Zoom solution: Promoting effective cross-ideological communication online
Source: PLoS One. 2022 Jul 20;17(7):e0270355. doi: 10.1371/journal.pone.0270355 (PMC9299349; doi:10.1371/journal.pone.0270355)
Supplement: S1 Appendix — (DOCX) [file pone.0270355.s001.docx]

**Supplementary Information**

The Zoom solution: Promoting effective cross-ideological communication online

Ashley L. Binnquist, Stephanie Y. Dolbier, Macrina C. Dieffenbach, and Matthew D. Lieberman

*Matthew D. Lieberman

Email: lieber@ucla.edu

**This PDF file includes:**

Scheduling and confederates, p. 2

Tables S1 to S7, p. 3-9

Description of top quartile analysis, p. 10

Tables S8 to S16, p.11-21

Recruitment Materials, p. 22

Survey materials, p. 23-34

**Scheduling, confederates, and coder training**

Four participants were scheduled for each experimental session based on availability and matched on issue, with two on each side of the issue. To accommodate for no-shows, two confederates were hidden in a breakout room of the Zoom call, ready to pose as participants. If a participant did not show, one of the participants on the other side of the issue was sent home, and confederates filled the role of the no-show and the sent-home participants. If two participants did not show, but were on opposing sides of the issue, confederates filled the role; otherwise, the session was rescheduled. As such, sessions were either run with 4 real participants, or with 2 real participants and two confederates (one real participant and confederate on each side of the issue).

Regardless of condition, all participants first had a conversation with an ingroup member. If confederates were used, each real participant’s ingroup conversation partner would be with a confederate. The CIC always consisted of two real participants talking to each other. In the *private* condition the instructions remained the same as when there were no confederates. However, in the *public* condition with confederates the participants were told that after the second conversation more instructions would follow. Once the CIC with real participants was over, one of the experimenters gave instructions that Pro 2 and Con 2 (the confederates) would be sent to a separate breakout room for a private conversation. Pro 1 and Con 1 (participants) were instructed to not leave the Zoom session as they would be given instructions for a post-study survey. The two confederates were moved to a breakout room as if another conversation were going to occur. After completion of the post-study survey participants were debriefed.

Confederates were extensively trained by watching videos from test sessions, mock conversations, and guidance on how to engage in the conversation without overbiasing the participant. At the start of the conversation, confederates were instructed to wait for the participant to speak or suggest the participant could state their opinion first. If the participant protested and prefer the confederate start the conversation the confederate was trained to give a similar opinion but not introduce new information. It was strongly emphasized that confederates avoid introducing new ideas throughout the conversation, and instead do their best to agree or expand upon the ideas the participant brought up during the ingroup conversation.

Coders were instructed to remain as objective as possible and to avoid letting their own opinion on the issue influence their ratings of the conversations. Before rating the videos, coders were trained by first rating test conversations from a pilot study. To help orient the coders when rating the conversations, brief qualitative descriptions were given for what type of behavior may occur in each of the five sub-divisions within the conflict rating scale. Lastly, to avoid order and sequence effects, each coder was given a unique randomized list of the conversations.

**Table S1. Demographic characteristics of participants**

|  | *n = 122* | Sample % | *n = 106* ^a^ | Sample % |
| --- | --- | --- | --- | --- |
| Gender |  |  |  |  |
| Female | 59 | 48.36 | 52 | 49.06 |
| Male  Non-binary | 59  4 | 48.36  3.28 | 52  2 | 49.06  1.89 |
| Race/Ethnicity |  |  |  |  |
| African American/black | 12 | 9.84 | 11 | 10.38 |
| Asian | 9 | 7.38 | 8 | 7.55 |
| Hispanic/Latinx  White  Mixed race ^b^ | 11  79  9 | 9.02  64.75  7.38 | 9  69  7 | 8.49  65.09  6.60 |
| Other/Prefer not to answer | 2 | 1.64 | 2 | 1.89 |
| Age |  |  |  |  |
| Range (18-40) | 30.36 $\pm$5.65 |  | 30.51 $\pm$5.7 |  |

*Note.* Age reported mean and standard deviation.

^a^ Demographics for participants who completed all pre-study forecast questions.

ᵇ Mixed race participants included: African American/black and white (1), Asian and white (3), Hispanic/Latinx and Pacific Islander (1), and American Indian/Alaskan Native and White (4).

**Table S2. Individual specific CIC questions asked of participants and coders**

| Pre-CIC Self-report | Post-CIC Self-report | Coder prompt | Abbreviation |
| --- | --- | --- | --- |
| What percentage of the time would you be in conflict with the person of the opposite opinion during this conversation | What percentage of the time would you say that you two were in conflict | What percentage of the time would you say that the participant was getting along with their partner of the opposite opinion | Conflict ^a^ |
| What percentage of the time would you be getting along with the person of the opposite opinion during this conversation | What percentage of the time would you say that you two were getting along | What percentage of the time would you say the participant was in conflict with their partner of the opposite opinion | Get along ^a^ |
| I feel the interaction would be difficult to get through | I felt the interaction was difficult to get through | The interaction looked difficult to get through for them | Difficult |
| I feel this interaction would be stressful | I feel this interaction was very stressful | The interaction looked very stressful for them | Stress |
| I think I could respect their opinions | I could respect their opinions | They could respect their partners opinions | Respect |
| I feel this interaction would be enjoyable | I felt the interaction was enjoyable | It looked like they enjoyed the interaction | Enjoy |
| I feel the interaction would be difficult to get through | I liked them as a person | They liked them as a person | Like Partner |
| I believe their ideas would be convincing | I thought their ideas were convincing | They looked convinced by their partners statements | Convince |
| N/A | I listened carefully to them | They listened carefully | Listened |
| I would feel comfortable in this interaction | I felt comfortable in the interaction | They looked comfortable in the interaction | Comfort |
| N/A | I was motivated to engage in the discussion | They were motivated to engage in the discussion | Motivated |
| N/A | I dominated the conversation | They dominated the conversation | Dominated |
| N/A | I felt like I was able to say what I wanted | They looked like they were able to say what they wanted | Spoke openly |
| I believe their ideas would be valid | I thought their ideas were valid | N/A | Valid |
| I believe their statements would be driven by emotion | Their statements were driven by emotion | N/A | Emotional |
| I believe their statements would be driven by logic | Their statements were driven by logic | N/A | Logical |
| I would be afraid of being judged by the others present | I felt judged by this person | N/A | Judged |
| How informed do you feel about the issue? | Same as pre-CIC | N/A | Informed ^b^ |
| How much do you care about the issue? | Same as pre-CIC | N/A | Caring ^b^ |
| How informed do you feel about the issue? | Same as pre-CIC | N/A | Importance ^b^ |
| How much more correct is your opinion on this issue compared to the opposite opinion? | Same as pre-CIC | After the conversation, how much more correct do you think the participant believes their opinion on this issue is compared to the opposite opinion | Correctness ^c^ |
| Below you will rate how you feel, favorably or unfavorably, towards people with specific opinions on [Issue based statement] | Same as pre-CIC | N/A | Outgroup Warmth ^d^ |
| Please indicate how strongly you agree with the following [Issue based statement] | Same as pre-CIC | What do you believe the participant’s attitude was at the [START and END] of the conversation for the [Issue based statement] | Attitude Extremity ^e^ |

*Note.* Questions are written as they were asked of individuals in the pre-study survey, post-study survey after the CIC, and for the coder survey. All variables, unless noted were measured from 1 (strongly disagree) to 7 (strongly agree).

^a^ Assessed on a 0-100% scale.

^b^ Measured on a scale of 1 (not at all) to 4 (extremely).

^c^ Measured on a scale of 1 (No more correct than the other opinion) to 4 (Totally correct-mine is the only correct opinion).

^d^ Measured on a scale of 1 (extremely unfavorable) to 7 (extremely favorable).

^e^ Attitude was collapsed at the mid-point for extremity to ignore ideological leaning in analysis.

**Table S3. Coder specific questions about the CIC for the individual**

| Coder prompt | Abbreviation |
| --- | --- |
| They looked frustrated in the interaction | Frustrated |
| They used an inflammatory tone | Inflammatory |
| They seemed to be arguing in bad faith | Bad faith |
| How much do you feel that they were privately disagreeing in their head, but not outwardly showing it? | Private disagree |
| How much do you feel that they were outwardly expressing disagreement? | Outward disagree |
| They heatedly disagreed | Heated |
| They instigated conflict | Instigated |
| They hesitated to express disagreement | Hesitated |
| They looked nervous in the interaction | Nervous |

*Note.* Conflict and getting along were assessed on a 0-100% scale. All other variables were measured from 1 (strongly disagree) to 7 (strongly agree).

**Table S4. Coder specific questions about the CIC as a group**

| Coder prompt | Abbreviation |
| --- | --- |
| Overall, how much hidden conflict was there in this conversation? | Hidden conflict |
| Overall, how much overt conflict was there in this conversation? | Overt conflict |
| How would you describe the conversation in terms of the type of conflict in the conversation, if any? | Overall conflict |
| How would you describe the conversation in terms of how much participants agreed with each other? | Agreement |
| How much do you agree that the two participants seemed like very different types of people? | Different |

*Note.* Conflict and getting along were assessed on a 0-100% scale. All other variables were measured from 1 (strongly disagree) to 7 (strongly agree).

**Table S5.** **Dependent samples t-tests for Participants’ Forecasts vs Experience in the CIC (n = 120).**

|  | Forecasted | | Experienced | | *t* | *p* | Cohen’s *d* | 95% CI | |
| --- | --- | --- | --- | --- | --- | --- | --- | --- | --- |
|  | *M* | *SD* | *M* | *SD* |  |  |  |  |  |
| Conflict ^a^ | 54.44 | 25.54 | 23.26 | 26.95 | 9.85 | <.001 | -1.19 | | [-37.5, -24.9] |
| Getting along ^a^ | 48.26 | 23.72 | 78.82 | 24.10 | 10.15 | <.001 | 1.28 | | [24.6, 36.5] |
| Enjoy | 3.60 | 1.59 | 5.72 | 1.39 | 13.44 | <.001 | 1.42 | | [1.80, 2.43] |
| Stress | 4.32 | 1.66 | 2.47 | 1.66 | 11.04 | <.001 | -1.11 | | [-2.19, -1.53] |
| Comfort ^b^ | 4.25 | 1.64 | 5.70 | 1.32 | 9.44 | <.001 | 0.97 | | [1.14, 1.75] |
| Difficult | 4.08 | 1.66 | 2.65 | 1.65 | 7.94 | <.001 | -0.86 | | [-1.79, -1.08] |
| Like partner ^a^ | 3.70 | 1.30 | 5.66 | 1.31 | 13.48 | <.001 | 1.50 | | [1.67, 2.25] |
| Respect ^a^ | 4.82 | 1.63 | 5.95 | 1.25 | 7.29 | <.001 | 0.78 | | [0.82, 1.44] |
| Valid ^a^ | 4.22 | 1.56 | 5.42 | 1.49 | 7.74 | <.001 | 0.79 | | [0.89, 1.51] |
| Convinced ^a^ | 3.30 | 1.70 | 4.30 | 1.89 | 5.44 | <.001 | 0.56 | | [0.64, 1.36] |
| Emotional ^a^ | 5.24 | 1.33 | 4.41 | 1.94 | 4.06 | <.001 | -0.50 | | [-1.24, -0.42] |
| Logical ^c^ | 3.37 | 1.61 | 4.57 | 1.73 | 7.05 | <.001 | 0.72 | | [0.86, 1.54] |
| Judge | 3.35 | 1.91 | 2.69 | 1.74 | 2.86 | .005 | -0.36 | | [-1.05, -0.19] |

*Note.* The mean difference is reported as experienced minus forecasted. Conflict and getting along were assessed on a 0-100% scale. All other variables were measured from 1 (strongly disagree) to 7 (strongly agree). Exact wording of questions is reported in Table S2.

^a^ Sample size is *n =* 106 due to an administrative error.

^b^ Sample size is *n =* 119 due to an administrative error.

^c^ Sample size is *n* =105 due to an administrative error.

**Table S6.** **Dependent samples t-tests for Participants’ Forecasts vs Experience in the CIC (n = 120) separated by condition.**

|  | Forecasted | | Experienced | | *t* | *p* | Cohen’s *d* | 95% CI |
| --- | --- | --- | --- | --- | --- | --- | --- | --- |
|  | *M* | *SD* | *M* | *SD* |  |  |  |  |
| Conflict (*Private*) | 56.90 | 26.61 | 19.07 | 25.23 | 9.58 ^a^ | <.001 | -1.22 | [-45.74, -29.93] |
| Conflict (*Public*) | 51.24 | 23.98 | 28.74 | 28.39 | 4.57 ^a^ | <.001 | -0.86 | [-32.42, -12.58] |
| Get along (*Private*) | 46.35 | 24.63 | 82.25 | 22.87 | 9.12 ^a^ | <.001 | 1.51 | [28.02, 43.78] |
| Get along (*Public*) | 50.76 | 22.50 | 74.35 | 25.17 | 5.24 ^a^ | <.001 | 0.99 | [14.52, 32.65] |
| Enjoy (*Private*) | 3.62 | 1.67 | 5.83 | 1.42 | 9.05 | <.001 | 1.43 | [1.73, 2.71] |
| Enjoy (*Public*) | 3.58 | 1.52 | 5.60 | 1.36 | 10.12 | <.001 | 1.40 | [1.62, 2.42] |
| Stress (*Private*) | 4.20 | 1.79 | 2.17 | 1.55 | 8.64 | <.001 | -1.21 | [-2.50, -1.56] |
| Stress (*Public*) | 4.45 | 1.52 | 2.77 | 1.72 | 7.00 | <.001 | -1.04 | [-2.16, -1.20] |
| Comfort (*Private*) | 4.28 | 1.81 | 5.85 | 1.38 | 6.14 ^b^ | <.001 | 0.98 | [1.06, 2.08] |
| Comfort (*Public*) | 4.22 | 1.46 | 5.54 | 1.26 | 7.86 ^b^ | <.001 | 0.97 | [0.99, 1.66] |
| Difficult (*Private*) | 3.93 | 1.72 | 2.30 | 1.61 | 6.50 | <.001 | -0.98 | [-2.14, -1.13] |
| Difficult (*Public*) | 4.23 | 1.60 | 3.00 | 1.64 | 4.77 | <.001 | -0.76 | [-0.72, 1.75] |
| Like partner (*Private*) | 3.65 | 1.42 | 5.82 | 1.32 | 10.31^a^ | <.001 | 1.58 | [1.75, 2.59] |
| Like partner (*Public*) | 3.76 | 1.12 | 5.46 | 1.28 | 8.98 ^a^ | <.001 | 1.41 | [1.32, 2.08] |
| Respect (*Private*) | 4.65 | 1.84 | 6.05 | 1.29 | 6.38 ^a^ | <.001 | 0.88 | [0.96, 1.84] |
| Respect (*Public*) | 5.04 | 1.30 | 5.83 | 1.20 | 3.80 ^a^ | <.001 | 0.63 | [0.37, 1.20] |
| Valid (*Private*) | 4.08 | 1.66 | 5.25 | 1.65 | 5.45 ^a^ | <.001 | 0.71 | [0.74, 1.60] |
| Valid (*Public*) | 4.39 | 1.42 | 5.63 | 1.22 | 5.52 ^a^ | <.001 | 0.94 | [0.79, 1.69] |
| Convinced (*Private*) | 3.28 | 1.75 | 4.40 | 1.87 | 4.52 ^a^ | <.001 | 0.62 | [0.62, 1.61] |
| Convinced (*Public*) | 3.33 | 1.66 | 4.17 | 1.94 | 3.07 ^a^ | .004 | 0.47 | [0.29, 1.40] |
| Emotional (*Private*) | 5.60 | 1.22 | 4.82 | 1.94 | 2.79 ^a^ | .007 | -0.48 | [-1.34, -0.22] |
| Emotional (*Public*) | 4.76 | 1.34 | 3.87 | 1.83 | 2.97 ^a^ | .005 | -0.55 | [-1.50, -0.29] |
| Logical (*Private*) | 3.30 | 1.68 | 4.50 | 1.79 | 4.82 ^c^ | <.001 | 0.69 | [0.70, 1.70] |
| Logical (*Public*) | 3.47 | 1.52 | 4.67 | 1.65 | 5.42 ^c^ | <.001 | 0.76 | [0.75, 1.65] |
| Judged (*Private*) | 3.37 | 1.95 | 2.48 | 1.60 | 2.82 | .006 | -0.50 | [-1.51, 0.26] |
| Judged (*Public*) | 3.33 | 1.88 | 2.90 | 1.86 | 1.16 | .250 | -0.23 | [-0.94, 0.25] |

*Note.* Conflict and getting along were assessed on a 0-100% scale. All other variables were measured from 1 (strongly disagree) to 7 (strongly agree). Exact wording of questions is reported in Table S2.

^a^ Sample size is *n =* 106 due to an administrative error.

^b^ Sample size is *n =* 119 due to an administrative error.

^c^ Sample size is *n* =105 due to an administrative error.

**Table S7.** **Frequency of disposition for Pre-CIC and post-CIC forecasted/experienced variables (n = 120).**

|  | Pre-CIC disposition | | | | Post-CIC disposition | | | | | | |  | | | Pairwise Comparisons | | | | |  |
| --- | --- | --- | --- | --- | --- | --- | --- | --- | --- | --- | --- | --- | --- | --- | --- | --- | --- | --- | --- | --- |
|  | Negative | Neutral | Positive | | | Negative | | Neutral | | Positive | | |  |  | | NT vs PS | | NG vs PS | | |
|  | N (%) | N (%) | N (%) | | | N (%) | | N (%) | | N (%) | | | $\chi^{2}$ | *p* | | *Adj. p* | | *Adj. p* | | |
| *Negative Valence Variables* | | | |  | | |  | |  |  |  | | |  | | |  | |  |  |
| Conflict ^a^ | 51 (48.1) | 17 (16.0) | 38 (35.9) | | | 14 (13.2) | | 4 (3.8) | | 88 (83.0) | | | 48.95 | <.001 | | <.001 | | <.001 | | |
| Emotional ^a^ | 77 (72.6) | 20 (18.9) | 9 (8.5) | | | 48 (45.3) | | 6 (5.7) | | 52 (49.1) | | | 44.58 | <.001 | | <.001 | | <.001 | | |
| Stress | 65 (54.2) | 22 (18.3) | 33 (27.5) | | | 18 (15.0) | | 7 (5.8) | | 95 (79.2) | | | 64.40 | <.001 | | <.001 | | <.001 | | |
| Difficult | 63 (52.5) | 14 (11.7) | 43 (35.8) | | | 24 (20.0) | | 9 (7.5) | | 87 (72.5) | | | 33.46 | <.001 | | .063 | | <.001 | | |
| Judged | 40 (33.3) | 16 (13.3) | 64 (53.3) | | | 25 (21.2) | | 7 (5.9) | | 86 (72.9) | | | 10.19 | .006 | | .087 | | .050 | | |
| *Positive Valence Variables* | | | |  | | |  | |  |  |  | | |  | | |  | |  |  |
| Getting along ^a^ | 45 (42.5) | 17 (16.0) | 44 (41.5) | | | 13 (12.3) | | 2 (1.9) | | 91 (85.8) | | | 45.86 | <.001 | | <.001 | | <.001 | | |
| Enjoy | 59 (49.2) | 25 (20.8) | 36 (30.0) | | | 9 (7.5) | | 8 (6.7) | | 103 (85.8) | | | 77.82 | <.001 | | <.001 | | <.001 | | |
| Convinced ^a^ | 56 (52.8) | 22 (20.8) | 28 (26.4) | | | 43 (40.6) | | 8 (7.5) | | 55 (51.9) | | | 17.02 | <.001 | | .001 | | .010 | | |
| Comfort ^b^ | 47 (39.5) | 13 (10.9) | 59 (49.6) | | | 12 (10.1) | | 5 (4.2) | | 102 (85.7) | | | 35.80 | <.001 | | .023 | | <.001 | | |
| Logical ^c^ | 53 (50.5) | 22 (21.0) | 30 (28.5) | | | 5 (4.8) | | 1 (1.0) | | 99 (94.3) | | | 95.81 | <.001 | | <.001 | | <.001 | | |
| Like partner ^a^ | 33 (31.3) | 56 (52.8) | 17 (16.0) | | | 5 (4.7) | | 11 (10.4) | | 90 (84.9) | | | 100.66 | <.001 | | <.001 | | <.001 | | |
| Valid ^a^ | 30 (28.3) | 22 (20.8) | 54 (50.9) | | | 15 (14.2) | | 6 (5.7) | | 85 (80.2) | | | 21.06 | <.001 | | <.001 | | .006 | | |
| Respect ^a^ | 17 (16.0) | 15 (14.2) | 74 (69.8) | | | 7 (6.6) | | 5 (4.7) | | 94 (88.7) | | | 11.55 | .003 | | .051 | | .075 | | |

*Note.* Chi-square analysis of disposition when comparing pre-CIC to post-CIC was significant for all variables tested. Post-hoc pairwise comparisons, shown with Bonferroni adjusted p-value, show the change in proportion was driven by the change in proportion in the negative versus positive and neutral vs positive dispositions, such that the proportion of the positive disposition range became larger post-CIC. The negative vs neutral pairwise comparison is not shown as none of the pairwise comparisons approached significance. Negative disposition = NG, neutral disposition = NT, and positive disposition = PS. Exact wording of questions is reported in Table S2.

^a^ Sample size is *n =* 106 due to an administrative error.

^b^ Sample size is *n =* 119 due to an administrative error.

^c^ Sample size is *n* =105 due to an administrative error.

**Affective Forecasting analyses for participants in the top quartile of experienced conflict**

One possible explanation for the large inaccuracies in participants’ forecasts may be that some participants may have had polite, nonconfrontational, and reasonable conversations that are atypical of the norm. To test this explanation, we look at individuals whose experienced conflict was in the top 25% of participants. These individuals experienced conflict in-line with their original expectations, and yet they still exhibited inaccurate forecasts in a number of domains (see Table S5). These ‘high conflict’ participants still found themselves having a more positive, and less negative experience in several domains. While not all factors remain significant, the robustness of more general affective experiences such as enjoyment, stress, and liking are remarkable, especially given the low statistical power that comes with the reduced sample size of *n* =26. Among the other factors, the mean differences show the same general direction as in the total sample, so it is possible that with a larger sample size of these ‘high conflict’ conversations these effects could be confirmed. This suggests that even for people who have a more conflict-filled interaction in line with their original expectations, the experience is still more positive than expected.

**Table S8. Dependent samples t-tests for Forecasts vs Experience in the CIC for participants in the top 25% of experienced conflict (n = 26).**

|  | Forecasted | | Experienced | | *t* | *p* | | Cohen’s *d* | | 95% CI |
| --- | --- | --- | --- | --- | --- | --- | --- | --- | --- | --- |
|  | *M* | *SD* | *M* | *SD* |  |  |  |  | |  |
| Conflict | 61.46 | 27.00 | 63.50 | 21.81 | 0.41 | .688 | -0.08 | | [-8.31, 12.39] | |
| Getting along | 45.46 | 25.77 | 49.58 | 26.58 | 0.67 | .510 | 0.16 | | [-8.55, 16.78] | |
| **Enjoy** | 3.65 | 1.98 | 5.38 | 1.70 | 4.11 | <.001 | 0.94 | | [0.74, 2.32] | |
| **Stress** | 4.08 | 2.06 | 2.92 | 1.98 | 2.72 | .012 | -0.57 | | [-1.78, -0.22] | |
| **Comfort** ^a^ | 4.32 | 1.82 | 5.36 | 1.66 | 3.11 | .005 | 0.60 | | [0.35, 1.73] | |
| Difficult | 3.96 | 1.99 | 3.04 | 1.75 | 1.96 | .061 | -0.49 | | [-1.69, 0.03] | |
| **Like partner** | 3.65 | 1.47 | 4.96 | 1.78 | 4.25 | <.001 | 0.80 | | [0.68, 1.94] | |
| Respect | 4.73 | 1.56 | 5.27 | 1.61 | 1.90 | .070 | 0.34 | | [-0.05, 1.12] | |
| Valid | 3.88 | 1.66 | 4.58 | 1.72 | 2.06 | .050 | 0.41 | | [-0.01, 1.39] | |
| Convinced | 3.08 | 1.83 | 3.23 | 1.77 | 0.55 | .589 | 0.08 | | [-0.43, 0.73] | |
| Emotional | 5.15 | 1.54 | 4.88 | 1.93 | 0.74 | .464 | -0.15 | | [-1.01, 0.48] | |
| **Logical** | 3.04 | 1.66 | 3.73 | 1.91 | 2.09 | .047 | 0.39 | | [0.01, 1.38] | |
| Judged | 2.96 | 1.82 | 3.50 | 1.90 | 1.34 | .191 | 0.29 | | [-0.29, 1.36] | |

*Note.* The mean difference is reported as experienced minus forecasted. Conflict and getting along were assessed on a 0-100% scale. All other variables were measured from 1 (strongly disagree) to 7 (strongly agree). Exact wording of questions is reported in Table S2. Significance, indicated by bolded font, was reported at an alpha level of 0.05.

^a^ Sample size is *n* = 25 due to an administrative error.

**Table S9. Dependent samples t-Tests for Issue-based attitudes and feelings, pre- to post-experiment (n = 122)**

|  | Pre-CIC | | Post-CIC | | *t* | *p* | Cohen’s *d* | | | 95% CI |
| --- | --- | --- | --- | --- | --- | --- | --- | --- | --- | --- |
|  | *M* | *SD* | *M* | *SD* |  |  |  | | |  |
| Attitude Extremity | 2.36 | 0.75 | 2.15 | 0.78 | 2.49 | .014 | | -0.27 | [-0.38, -0.04] | |
| Coder rated | 2.00 | 0.60 | 1.80 | 0.71 | 5.46 | <.001 | | -0.30 | [-0.27, -0.12] | |
| Informed | 2.79 | 0.78 | 3.10 | 0.64 | 4.88 | <.001 | | 0.43 | [0.19, 0.44] | |
| Caring | 3.01 | 0.90 | 3.24 | 0.72 | 3.01 | .003 | | 0.28 | [0.08, 0.38] | |
| Importance | 3.12 | 0.92 | 3.35 | 0.73 | 2.74 | .007 | | 0.28 | [0.06, 0.40] | |
| Correctness | 2.56 | 0.86 | 2.25 | 0.94 | 3.69 | <.001 | | -0.34 | [-0.47, -0.14] | |
| Outgroup Warmth | 2.67 | 1.24 | 3.30 | 1.34 | 4.56 | <.001 | | 0.49 | [0.36, 0.90] | |

*Note.* Coders only rated attitude for pre and post. The mean difference is reported as post-CIC minus pre-CIC. All variables were measured from 1 (strongly disagree) to 7 (strongly agree). Exact wording of questions is reported in Table S2.

**Table S10. Dependent samples t-Tests for Issue-based attitudes and feelings, pre- to post-experiment (n = 122)**

|  | Pre-CIC | | Post-CIC | | *t* | *p* | | Cohen’s *d* | | 95% CI |
| --- | --- | --- | --- | --- | --- | --- | --- | --- | --- | --- |
|  | *M* | *SD* | *M* | *SD* |  |  |  |  | |  |
| Attitude Extremity (*Private*) | 2.37 | 0.78 | 2.12 | 0.83 | 1.79 | .079 | -0.31 | | [-0.53, 0.03] | |
| Attitude Extremity (*Public*) | 2.35 | 0.73 | 2.18 | 0.74 | 1.75 | .086 | -0.23 | | [-0.38, 0.03] | |
| Coder rated (*Private*) | 2.01 | 0.59 | 1.75 | 0.73 | 5.42 | <.000 | -0.39 | | [-0.35, -0.16] | |
| Coder rated (Public) | 1.99 | 0.62 | 1.85 | 0.69 | 2.58 | .012 | -0.21 | | [-0.24, 0.03] | |
| Informed (*Private*) | 2.95 | 0.83 | 3.15 | 0.66 | 2.56 | .013 | 0.27 | | [0.04, 0.36] | |
| Informed (*Public*) | 2.63 | 0.71 | 3.05 | 0.61 | 4.23 | <.001 | 0.63 | | [0.22, 0.62] | |
| Care (*Private*) | 2.93 | 1.01 | 3.18 | 0.77 | 2.04 | .046 | 0.28 | | [0.00, 0.50] | |
| Care (*Public*) | 3.08 | 0.80 | 3.29 | 0.66 | 2.27 | .027 | 0.29 | | [0.02, 0.39] | |
| Importance (*Private*) | 2.90 | 1.08 | 3.25 | 0.73 | 2.46 | .017 | 0.38 | | [0.07, 0.63] | |
| Importance (*Public*) | 3.34 | 0.68 | 3.45 | 0.72 | 1.26 | .211 | 0.16 | | [-0.07, 0.29] | |
| Correctness (*Private*) | 2.53 | 0.85 | 2.22 | 1.01 | 2.87 | .006 | -0.33 | | [-0.54, -0.10] | |
| Correctness (*Public*) | 2.58 | 0.88 | 2.29 | 0.88 | 2.37 | .021 | -0.33 | | [-0.54, 0.05] | |
| Outgroup Warmth (*Private*) | 2.52 | 1.27 | 3.37 | 1.44 | 4.83 | <.001 | 0.63 | | [0.50, 1.20] | |
| Outgroup Warmth (*Public*) | 2.82 | 1.21 | 3.24 | 1.24 | 2.00 | .051 | 0.34 | | [-0.00, 0.84] | |

*Note.* Coders only rated attitude for pre and post. The mean difference is reported as post-CIC minus pre-CIC. All variables were measured from 1 (strongly disagree) to 7 (strongly agree). Exact wording of questions is reported in Table S2.

**Table S11. Independent samples t-Tests for condition-based differences in the CIC (n = 122)**

|  | Private | | Public | | *t* | *p* | Cohen’s *d* | | 95% CI |
| --- | --- | --- | --- | --- | --- | --- | --- | --- | --- |
|  | *M* | *SD* | *M* | *SD* |  |  |  |  |  |
| **Conflict** | 19.07 | 25.23 | 29.27 | 30.06 | 2.03 | .044 | 0.37 | [0.27, 20.14] | |
| **Coder rated** | 18.05 | 18.02 | 25.44 | 19.64 | 2.17 | .032 | 0.39 | [0.64, 14.14] | |
| **Getting along** | 82.25 | 22.87 | 72.81 | 28.00 | 2.04 | .043 | -0.37 | [-18.60, -0.29] | |
| **Coder rated** | 85.34 | 14.69 | 76.02 | 16.67 | 3.28 | .001 | -0.59 | [-14.95, -3.70] | |
| **Difficult** | 2.30 | 1.61 | 3.02 | 1.63 | 2.44 | .016 | 0.44 | [0.14, 1.30] | |
| **Coder rated** | 2.24 | 0.80 | 2.56 | 0.83 | 2.17 | .032 | 0.39 | [0.03, 0.61] | |
| Stress | 2.17 | 1.55 | 2.74 | 1.71 | 1.95 | .054 | 0.35 | [-0.01, 1.16] | |
| **Coder rated** | 2.11 | 0.71 | 2.56 | 0.78 | 3.40 | .001 | 0.62 | [0.19, 0.72] | |
| Respect | 6.05 | 1.29 | 5.76 | 1.41 | 1.19 | .236 | -0.22 | [-0.78, 0.19] | |
| **Coder rated** | 5.83 | 0.89 | 5.41 | 0.91 | 2.55 | .012 | -0.46 | [-0.74, -0.09] | |
| Enjoy | 5.83 | 1.42 | 5.60 | 1.36 | 0.92 | 0.36 | -0.17 | [-0.74, 0.27] | |
| **Coder rated** | 5.44 | 0.95 | 4.82 | 1.00 | 3.55 | .001 | -0.64 | [-0.97, -0.28] | |
| Like Partner | 5.82 | 1.32 | 5.40 | 1.36 | 1.70 | .091 | -0.31 | [-0.89, 0.07] | |
| **Coder rated** | 5.83 | 0.83 | 5.41 | 0.91 | 2.55 | .012 | -0.47 | [-0.74, -0.09] | |
| Convince | 4.40 | 1.87 | 4.31 | 1.98 | 0.27 | .789 | -0.05 | [-0.78, 0.60] | |
| **Coder rated** | 4.93 | 1.26 | 4.31 | 1.35 | 2.61 | .010 | -0.47 | [-1.09, -0.15] | |
| Listened | 6.40 | 0.87 | 6.35 | 0.55 | 0.34 | .732 | -0.07 | [-0.31, 0.22] | |
| **Coder rated** | 6.08 | 0.41 | 5.89 | 0.47 | 2.36 | .020 | -0.43 | [-0.35, -0.03] | |
| Comfort | 5.85 | 1.38 | 5.52 | 1.25 | 1.40 | .164 | -0.25 | [-0.81, 0.14] | |
| **Coder rated** | 5.58 | 0.89 | 5.22 | 0.89 | 2.24 | .027 | -0.40 | [-0.68, -0.04] | |
| Motivated | 6.40 | 0.72 | 6.29 | 0.66 | 0.88 | .383 | -0.16 | [-0.36, 0.14] | |
| Coder rated | 6.08 | 0.76 | 5.83 | 0.69 | 1.86 | .065 | -0.34 | [-0.50, 0.02] | |
| Dominated | 3.23 | 1.54 | 3.66 | 1.67 | 1.47 | .144 | 0.27 | [-0.15, 1.00] | |
| Coder rated | 3.02 | 0.90 | 3.00 | 1.00 | 0.11 | .908 | -0.02 | [-0.36, 0.32] | |
| Spoke openly | 6.30 | 0.94 | 6.13 | 1.14 | 0.87 | .385 | -0.16 | [-0.55, 0.21] | |
| Coder rated | 3.02 | 0.90 | 3.00 | 1.00 | 0.12 | .908 | -0.02 | [-0.36, 0.32] | |

*Note.* All but one of the coder rated differences of condition follow the same trend of self-reported differences, that is more negativity and less positivity in the *public* vs. *private* condition. Significance, indicated by bolded font, was reported at an alpha level of 0.05. Exact wording of questions is reported in Table S2.

**Table S12. Independent samples t-Tests for unique individual coder rated condition-based differences in the CIC (n = 122)**

|  | Private | | Public | | *t* | *p* | Cohen’s *d* | | 95% CI |
| --- | --- | --- | --- | --- | --- | --- | --- | --- | --- |
|  | *M* | *SD* | *M* | *SD* |  |  |  |  |  |
| **Frustrated** | 2.14 | 0.90 | 2.59 | 1.02 | 2.62 | .010 | 0.47 | [0.11, 0.80] | |
| **Inflammatory** | 1.35 | 0.54 | 1.60 | 0.76 | 2.05 | .043 | 0.38 | [0.01, 0.48] | |
| **Bad Faith** | 1.23 | 0.34 | 1.38 | 0.46 | 2.11 | .037 | 0.37 | [0.01, 0.30] | |
| **Private disagree** | 1.77 | 0.61 | 2.02 | 0.63 | 2.22 | .029 | 0.40 | [0.03, 0.47] | |
| Outward disagree | 2.91 | 0.74 | 3.13 | 0.89 | 1.48 | .140 | 0.27 | [-07, 0.51] | |
| Heated | 1.44 | 0.72 | 1.75 | 1.06 | 1.89 | .061 | 0.34 | [-0.02, 0.63] | |
| Instigated | 1.32 | 0.46 | 1.47 | 0.54 | 1.69 | .093 | 0.30 | [-0.3, 0.33] | |
| Hesitated | 2.26 | 0.64 | 2.41 | 0.72 | 1.21 | .227 | 0.22 | [-0.9, 0.39] | |
| Nervous | 2.30 | 0.88 | 2.50 | 0.79 | 1.34 | .181 | 0.24 | [-0.10, 0.50] | |

*Note.* Significance, indicated by bolded font, was reported at an alpha level of 0.05. Exact wording of questions is reported in Table S3.

**Table S13. Independent samples t-Tests for dyadic coder rated condition-based differences in the CIC (n = 61)**

|  | Private | | Public | | *t* | *p* | Cohen’s *d* | | 95% CI |
| --- | --- | --- | --- | --- | --- | --- | --- | --- | --- |
|  | *M* | *SD* | *M* | *SD* |  |  |  |  |  |
| **Hidden conflict** | 1.93 | 0.57 | 2.32 | 0.64 | 2.56 | .013 | 0.64 | [0.09, 0.70] | |
| Overt conflict | 2.03 | 0.87 | 2.26 | 0.97 | 0.98 | .331 | 0.25 | [-0.24, 0.70] | |
| Overall conflict | 2.85 | 1.21 | 3.49 | 1.55 | 1.79 | .079 | 0.46 | [-0.08, 1.35] | |
| Agreed | 4.86 | 1.62 | 4.11 | 1.67 | 1.77 | .083 | -0.45 | [-1.60, 0.10] | |
| Different people* | 3.81 | 1.21 | 4.06 | 1.23 | 0.78 | .438 | 0.20 | [-0.38, 0.87] | |

*Note.* Coders rated these questions based on the conversation between both participants, not the participants as individuals. As such the sample size is based on the number of conversations (n = 61). Significance, indicated by bolded font, was reported at an alpha level of 0.05. Exact wording of questions is reported in Table S4.

**Table S14. Mixed subjects Analyses of Variance for liking of conversation partner**

Within Subjects Effects

|  | Sum of Squares | *df* | Mean Square | *F* | *p* | η^2^ |
| --- | --- | --- | --- | --- | --- | --- |
| Partner | 6.399 | 1, 120 | 6.399 | 7.319 | 0.008 | .019 |
| Condition x Partner | 3.530 | 1, 120 | 3.530 | 4.038 | 0.047 | .011 |

*Note.* Type II Sum of Squares

Between Subjects Effects

|  | Sum of Squares | | *df* | Mean Square | *F* | *p* | η^2^ |
| --- | --- | --- | --- | --- | --- | --- | --- |
| Condition | 1.822 | 1, 120 | | 1.822 | 0.992 | 0.321 | .006 |

*Note.* Type II Sum of Squares

Simple comparisons paired samples t-Test

|  | Ingroup Partner | | CIC Partner | | *t* | *p* | Cohen’s *d* | 95% CI |
| --- | --- | --- | --- | --- | --- | --- | --- | --- |
|  | *M* | *SD* | *M* | *SD* |  |  |  |  |
| Private | 5.90 | 0.97 | 5.82 | 1.32 | 0.50 | .616 | -0.07 | [-0.25, 0.41] |
| **Public** | 5.97 | 0.94 | 5.40 | 1.36 | 3.26 | .002 | -0.49 | [0.22, 0.91] |

*Note.* In the *public* condition participants liked their ingroup partner more than the CIC partner, even after Bonferroni correction. No difference in liking of the conversation partner was found for the *private* condition.

Simple comparisons independent samples t-Test

|  | Private | | Public | | *t* | *p* | Cohen’s *d* | 95% CI |
| --- | --- | --- | --- | --- | --- | --- | --- | --- |
|  | *M* | *SD* | *M* | *SD* |  |  |  |  |
| Ingroup Partner | 5.90 | 0.97 | 5.97 | 0.94 | 0.39 | .696 | 0.07 | [-0.89, 0.07] |
| CIC partner | 5.82 | 1.32 | 5.40 | 1.36 | 1.70 | .091 | 0.31 | [-0.27, 0.41] |

*Note.* No significant difference between conditions for liking of the ingroup or CIC partner.

**Table S15. Intraclass correlation estimates for reliability for coder rated variables**

| *Variable* | *ICC* | *p* | 95% CI | *Variable* | *ICC* | *p* | 95% CI |
| --- | --- | --- | --- | --- | --- | --- | --- |
| Conflict Pro | 0.81 | <.001 | [0.72, 0.87] | Spoke openly Pro | 0.49 | <.001 | [0.27, 0.67] |
| Conflict Con | 0.83 | <.001 | [0.75, 0.89] | Spoke openly Con | 0.56 | <.001 | [0.35, 0.71] |
| Getting along Pro | 0.75 | <.001 | [0.64, 0.83] | Nervous Pro | 0.60 | <.001 | [0.43, 0.74] |
| Getting along Con | 0.80 | <.001 | [0.72, 0.87] | Nervous Con | 0.66 | <.001 | [0.50, 0.77] |
| Difficult Pro | 0.59 | <.001 | [0.41, 0.73] | Frustrated Pro | 0.77 | <.001 | [0.67, 0.85] |
| Difficult Con | 0.57 | <.001 | [0.38, 0.72] | Frustrated Con | 0.72 | <.001 | [0.60, 0.82] |
| Stress Pro | 0.52 | <.001 | [0.31, 0.69] | Hesitated Pro | 0.49 | <.001 | [0.26, 0.66] |
| Stress Con | 0.61 | <.001 | [0.43, 0.74] | Hesitated Con | 0.59 | <.001 | [0.40, 0.73] |
| Respect Pro | 0.74 | <.001 | [0.62, 0.83] | Heated Pro | 0.74 | <.001 | [0.63, 0.83] |
| Respect Con | 0.81 | <.001 | [0.72, 0.87] | Heated Con | 0.84 | <.001 | [0.77, 0.90] |
| Enjoy Pro | 0.82 | <.001 | [0.74, 0.88] | Instigate Pro | 0.53 | <.001 | [0.32, 0.69] |
| Enjoy Con | 0.78 | <.001 | [0.68, 0.85] | Instigate Con | 0.64 | <.001 | [0.48, 0.76] |
| Like Partner Pro | 0.79 | <.001 | [0.70, 0.86] | Inflame Pro | 0.63 | <.001 | [0.46, 0.76] |
| Like Partner Con | 0.78 | <.001 | [0.69, 0.86] | Inflame Con | 0.77 | <.001 | [0.67, 0.85] |
| Convince Pro | 0.82 | <.001 | [0.75, 0.88] | Bad Faith Pro | 0.44 | <.001 | [0.20, 0.64] |
| Convince Con | 0.85 | <.001 | [0.78, 0.90] | Bad Faith Con | 0.65 | <.001 | [0.49, 0.77] |
| Listened Pro | 0.27 | 0.045 | [-0.05, 0.52] | Private Disagree Pro | 0.66 | <.001 | [0.50, 0.77] |
| Listened Con | 0.57 | <.001 | [0.38, 0.72] | Private Disagree Con | 0.65 | <.001 | [0.49, 0.77] |
| Comfort Pro | 0.71 | <.001 | [0.58, 0.81] | Outward Disagree Pro | 0.71 | <.001 | [0.58, 0.81] |
| Comfort Con | 0.71 | <.001 | [0.59, 0.81] | Outward Disagree Con | 0.77 | <.001 | [0.67, 0.85] |
| Motivated Pro | 0.85 | <.001 | [0.78, 0.90] | Correct Pro | 0.74 | <.001 | [0.62, 0.83] |
| Motivated Con | 0.65 | <.001 | [0.50, 0.77] | Correct Con | 0.82 | <.001 | [0.73, 0.88] |
| Dominated Pro | 0.54 | <.001 | [0.34, 0.70] | Attitude Pre Pro | 0.74 | <.001 | [0.63, 0.83] |
| Dominated Con | 0.71 | <.001 | [0.58, 0.81] | Attitude Pre Con | 0.71 | <.001 | [0.58, 0.81] |
| Direct Pro | 0.58 | <.001 | [0.39, 0.73] | Attitude Post Pro | 0.79 | <.001 | [0.69, 0.86] |
| Direct Con | 0.47 | <.001 | [0.24, 0.65] | Attitude Post Con | 0.86 | <.001 | [0.80, 0.91] |
| Hidden Conflict | 0.65 | <.001 | [0.50, 0.77] | Agreed | 0.91 | <.001 | [0.86, 0.94] |
| Overt Conflict | 0.84 | <.001 | [0.77, 0.90] | Different People | 0.76 | <.001 | [0.65, 0.84] |
| Overall Conflict | 0.83 | <.001 | [0.75, 0.89] |  |  |  |  |

*Note.* The majority of coder rated variables fell within the fair to excellent range with one exception of the variable that fell within the poor range. Exact wording of questions is reported in Tables S2-S4.

**Table S16. Post-CIC responses to the question: “This is the last question on the survey. Please use the box below to share any additional comments (optional).”**

**Comments related directly to the CIC (*n* = 37)**

| Attitude (Issue) | Response |
| --- | --- |
| *Con (Affirmative)* | This was a truly unique experience than anything else I've done on the mTurk platform and I'm very grateful that I was chosen to participate. I particularly enjoyed hearing another viewpoint that challenges my opinion that I hadn't really thought of before, and as such I think that a civil dialogue about these things can be incredibly productive, assuming that it isn't being drowned out by the twitter mob. |
| *Con (Affirmative)* | This was fun and very insightful! I wish we had gone on to the second conversation because I looked forward to hearing their point of view. [*Experimenter note*: the second conversation refers to the conversation they believed the confederates would have in private] |
| *Con (Affirmative)* | Thank you for this and giving me a chance to talk. I liked how everyone was my age ish and they were all nice. It seems like we all are in the middle-ish on some thing with this. |
| *Pro (Affirmative)* | Having the other two folks watching was really nerve-wracking, but occasionally I'd see my fellow pro nod and it made me feel reassured. This was really interesting and I wish I'd been able to have a follow-up conversation with my fellow Pro! |
| *Pro (Affirmative)* | The interaction was overall great! it would have been nice to have more prompts because there were awkward silences, especially since we do not know each other. Seems like this group was not very aggressive (thankfully) and respected everyones opinion and gave each other time to talk and express their opinions. |
| *Pro (Affirmative)* | Very interesting task. I will say that the second conversation (between opposing points of view) felt like it went by much faster! it was fun to listen to conflicting view points while both of us tried to express ourselves. The conversation was more stimulating. |
| *Pro (Affirmative)* | This was really great. I love having conversations like these. I would definitely be interested in doing this type of thing again. |
| *Pro (Business)* | The conversation was all together fascinating but I felt l was having to almost beg to elicit a response out of both conversations. |
| *Pro (Business)* | I felt this was a good experience on how to debate. Even though we had different ideas, I felt we were productive on how we expressed them. Wished we had a little more time. |
| *Con (Business)* | It didn't put more pressure on me to defend my opinion necessarily but it made me more nervous. [*Experimenter note*: previous question asked if having the ingroup present made them more nervous] |
| *Con (Business)* | I found it very interesting and informative and I enjoyed talking to other people with different perspectives on this topic as well as those who were in agreement with me. |
| *Pro (Father)* | It was an interesting experience! I didn't change my opinion or learn a whole lot necessarily, but I did expand my perspective a bit. |
| *Pro (Father)* | Thank you for this opportunity! I do think there was a great difference even between Con 1 and Con 2 in that Con was A LOT more domineering and steadfast with her opinion. Had I been asked to converse with her, I would have had trouble. Con 2 may have had a different opinion from mine, but I felt like she was more passive in comparison to Con and therefore I did not feel like it was as stressful as it was listening to Pro 1 vs Con 1. Pro 1 was stronger in saying his opinion, but he was still fair and validated Con, which I highly appreciated. If Pro 1 had a different opinion than I did, I think I would have been more likely to take his points into consideration more whereas Con 1 just seemed like she didn't want to hear the opposite view. I have to admit I was not surprised at the physical appearances of Con 1 and Con 2; I already got the sense both would be more feminist, which is why I consider myself to be an anomaly despite being a woman myself. I am under the impression I am in the minority with issues like these and am generally afraid to speak my mind because I feel so outnumbered. I felt at ease talking to Pro 1 for sure. As nervous as I was, I had a great time. Thank you to all! |
| *Con (Father)* | I feel the group that we were in was very open minded and not prone to confrontation. We all seemed to be able to nod in agreement with one another even if just to acknowledge that we accept the others opinion. Perhaps we were all moderate enough in our beliefs to seek common ground for discussion. I would be curious what this conversation would have been like if the opposing side was staunchly opposed to my point of view. Would their still be the same ability to find acceptance of opinion or would there be an enduring animosity from one or both sides? |
| *Con (Father)* | The conversation was a lot less hectic than I thought it would be, and I think that's because of how open the prompt truly was. If there was a more specific prompt, I feel the environment would be much different. |
| *Con (Father)* | I loved this session. Especially as stated earlier I had therapy concerning very deep topics this was relatively. I would have preferred some structure in the argument such as with evidence either from personal, social, or other forms that way it would lead the conversation in a way. However, overall I feel that our organic discussion from personal to legal view points were very valid and hopefully this helps in a way! |
| *Pro (Father)* | I felt I wanted to carry on with the conversation because I think they were pretty rational. I do not agree with what they had to say for the most part, but an additional conversation would be okay. |
| *Pro (Father)* | I felt the second conversation was a little more difficult because the person kept going on about personal experiences, not generalization or staying on topic. |
| *Pro (Father)* | The second person I spoke with was very friendly and disarming and this allowed me to feel more comfortable disagreeing with him. |
| *Pro (Gender)* | I think it is interesting to look at how an "audience" plays into how we converse or how it changes our views in dialogue. I think that my own views might be difficult to determine because I was trying to find middle ground with the person from conversation two. I felt that would be better than simply attacking their argument and would be a better tactic to convey my own ideas. |
| *Pro (Gender)* | I was not at all surprised that the other participant almost immediately brought up religion being one of her deciding factors on the topic, and honestly thats part of the reason (other than trying to be respectful) that I let her go first in the conversation. Being that in my opinion she is defending the wrong and hurtful side of the argument, I really wanted to see how she was going to defend her opinion as quickly as possible. |
| *Pro (Gender)* | The person from the second conversation was not who I was expecting. I pictured someone more stereotypically conservative, bigoted, and likely to recite propagandized arguments founded in emotion/fear/religion. Admittedly, I prejudged the participant before I met them. I was pleasantly surprised to meet someone much different, whose reasons for opposition were intelligent and founded in logic. The conversation did not convince me to change my opinion on the issue, but it did open me up to another perspective. |
| *Pro (Gender)* | Neither of the conversations was as difficult as I had imagined and neither held as extreme a view as I pictured. |
| *Con (Police)* | I enjoyed participating in this study, I wish there were more background questions for each participant so that the researchers could attempt to understand the reasoning of the participants. |
| *Con (Police)* | I think the conversations would go smoother if people were given "bullet points" to address. For example, instead of just broadly discussing the topic of police, we could also have bullet points that we could discuss one by one. There would only be a few important bullet points, such as "why are police important or unimportant in modern society?" Or, "Why is the funding of police problematic or beneficial to American citizens?" I feel that the conversations would be more organized this way, and people would not have the pressure of trying to create conversation material from off the top of their heads. |
| *Con (Police)* | Thank you for the chance to participate in this study, it was very informative and an overall good experience. :) |
| *Con (Police)* | I was nervous to come to the video call tonight but it was an interesting and civil conversation so I'm glad I did! |
| *Con (Police)* | Honestly, I enjoyed the second conversation more just because it was more intellectually stimulating to actually play devils advocate with each other respectfully rather than know we share roughly the same opinion. |
| *Con (Police)* | I really enjoyed this experience and honestly learned something that can and did make me reevaluate my stand. |
| *Pro (Police)* | First time ever doing this type of study. It was actually neat! I wasn't as nervous as I thought I would be. |
| *Pro (Police)* | everything about the survey was great! It was really interesting, even if I wasn't comfortable 100% of the time. |
| *Pro (Police)* | I really enjoyed this debate! I feel as if everyone where able to do this, we'd notice how similar our view points actually are, it's just a matter of how we solve those issues. If we stopped watching TV and started talking with our neighbors... we'd have a much more peaceful perspective. Thanks so much. |
| *Pro (Police)* | Honestly I thought it would be a lot more stressful than it was- it was fine. I think that they had valid points, concerns, and experiences that led them to believe that defunding the police was bad but I also felt like they were open to my opinions and point of view too. It was definitely an eye opener for me. |
| *Pro (Police)* | Having an audience that couldn't join in did increase the pressure a bit! But I think this research is important and I thank you for doing it. I would glady speak to either of my fellow conversationalist again. |
| *Pro (Police)* | The interaction with the first person was easy, we agreed on the basics of the topic and were pretty much just giving one another more dimension to our arguments.  The interaction with the second person was fun, because the person hadn't taken the opportunity to learn much about the subject. This isn't uncommon for any subject, people have lives to live - one of my hobbies just happens to be learning about any topic that I feel I may or should have an opinion on. The person was very, very open to what I had to say, and made several good, logical points in support of my argument. When she came into it, her opinion was almost entirely emotionally based on the scary way the phrase "defund the police" sounds, but when given information that she hadn't known before, she immediately used it to form a logical opinion. So, my answers to the above questions are heavily affected by her change of opinion.  I do feel my opinion on the subject is the correct one, but this is for a very general subject and I could see changing the specifics of my views based on a good, well researched argument. I could also see coming at extreme odds with someone with the same general opinion who disagreed on the specifics. |
| *Pro (Police)* | It was an interesting conversation. I appreciate being paired with a police officer. I am not sure that I was clear on my last line of my final thought. I meant to say that I agree that mental health care should be more funded and police less funded and it appeared that we agreed on that final point before the time was up. I regret not talking about race more directly especially since today is the anniversary of Tamir Rice's shooting. |
| *Pro (Police)* | It was less stressful talking to people who engaged more in conversation even if they had different beliefs which I found interesting. |

**General Comments related to the study (*n* = 39)**

| Attitude (Issue) | Response |
| --- | --- |
| *Con (Affirmative)* | I really enjoyed this interaction. |
| *Con (Affirmative)* | This was fun :) |
| *Con (Affirmative)* | Thanks for the work! |
| *Con (Affirmative)* | Thank you so much! |
| *Con (Affirmative)* | I thought this survey went very well and enjoyed talking to the other participants very much. Have a great day ahead of you! |
| *Con (Affirmative)* | Sorry for the tech issues! Cool study, though! |
| *Pro (Affirmative)* | I really enjoyed this experience. Thank you for the opportunity. I would love to do anymore surveys/research opportunities. Good luck on your projects and, if you are willing and able to share the final results/presentation without jeopardizing your experiment, I would love to see the final result. |
| *Pro (Affirmative)* | Really cool survey. Thank you for the opportunity and I hope the data provided is useful! |
| *Pro (Affirmative)* | This was a very interesting study! I enjoyed participating. Thanks :) |
| *Pro (Affirmative)* | none, thank you for having this interesting and awesome survey |
| *Pro (Affirmative)* | Great study, well set up and I had a great time! |
| *Pro (Affirmative)* | A very interesting project. Good luck with your analysis! |
| *Pro (Affirmative)* | Best of luck in your research! Thank you for the opportunity to participate. |
| *Pro (Business)* | interesting study. Thank you. |
| *Pro (Business)* | No comments at this time. Thank you for allowing me to participate! |
| *Pro (Business)* | None--Thank you for the opportunity! I enjoy this interview/interaction greatly! |
| *Con (Father)* | This was fun, thanks |
| *Con (Father)* | Great study I would love to be added to more studies . |
| *Con (Father)* | That was a lot of fun actually! Thanks for the opportunity! |
| *Con (Father)* | No extra comments. Thank you :) |
| *Pro (Father)* | Thank you |
| *Pro (Father)* | Fun study! |
| *Pro (Father)* | This is very interesting i hope to seeing more from you in the future. Thanks. |
| *Pro (Father)* | I really enjoyed getting to be a part of this study! I liked hearing views and opinions from the other side. Thank you! |
| *Con (Gender)* | Thank you I had a lot of fun. |
| *Con (Gender)* | Thank you for letting me be a part of this research. I hope that you have a great day, and stay safe! |
| *Con (Gender)* | This was sincerely one of the most interesting experiences I've had in a while, and I'm very thankful to have had this opportunity. |
| *Con (Gender)* | Thanks! This was very insightful! |
| *Con (Gender)* | Very interesting, I would be interested in other studies that you all put together. Thank you! |
| *Con (Gender)* | Thanks again for the opportunity. If you want to use my data or video please know I do accept that it can be used for presentation. |
| *Con (Police)* | That was really enjoyable. I hope we provided adequate data for the study. |
| *Con (Police)* | Interesting and enjoyable study. I hope being a test subject was helpful. |
| *Con (Police)* | Thank you for having me participate! best of luck with your research and study. |
| *Con (Police)* | Thank you for letting me be a part of it. I enjoyed it. |
| *Con (Police)* | None, this was awesome. I really enjoyed it. |
| *Pro (Police)* | I liked the survey! |
| *Pro (Police)* | That was great! Please feel free to contact me for any additional studies! Thanks! |
| *Pro (Police)* | I had a great experience doing this, and I'm glad that this study is being done! Thanks. |
| *Pro (Police)* | I greatly enjoyed this research study! Thank you for allowing me to participate and I hope your surveys went well on your end! |

**Comments related to the survey, technical issues, or suggestions (*n = 8*)**

| Attitude/Issue | Response |
| --- | --- |
| *Con (Affirmative)* | Your slide to the left and slide to the right question was very confusing. I’m not sure I answered it correctly. |
| *Pro (Affirmative)* | People given the choice are going to go with what they feel most comfortable with and that is people that come from the same background as them. We need pioneers to break barriers and pave the way for other backgrounds rather than cherry-picking and inserting people based on parameters that are irrelevant to the position. |
| *Con (Business)* | I wonder a bit how these conversations might have benefitted from slightly more structure, e.g., moderator questions. |
| *Con (Business)* | The two slider questions are kind of misleading to answer but other than that this has been fun, thank you so much! |
| *Pro (Father)* | There were times when I couldn't hear the person in the second conversation very well. |
| *Con (Gender)* | Adjust the wording on the scale in the survey "to the left of or to the right of" could be better thought you. And, in the discussion, it seemed odd that you would have only pair of opposition speak and the other listen. |
| *Pro (Gender)* | Those sliding scales about the furthest to the left and furthest to the right of your opinion are very confusing. Maybe I was just slow on the take, but I could not figure them out. |
| *Pro (Police)* | Maybe have the conversations last 15 minutes instead. |

**Recruitment Materials**

***Pre-screener mTurk post***

Project Name: Ideological Conversations Study (Part 1)

Title: Answer questions about your ideological opinions (WARNING: This HIT may contain adult content. Worker discretion is advised.)

Description: Report your opinions on various ideological and controversial issues (~5 minutes). You may qualify for a follow-up study.

keywords: survey, demographics, politics, opinions

***Pre-study mTurk post (only seen by eligible & scheduled participants)***

Project Name: Ideological Conversations Study (Part 2)

Title: Give your opinions, complete personality measures, and schedule a time to perform an interactive task with others who have similar and different views.

Description: You will answer demographic questions, give your opinions, and answer personality measures. This component takes ~20-30 mins. You will also sign up for a scheduled time to perform Part 3 of the experiment, which will involve answering a few questions and interacting via video chat with others who share similar and different ideological views from you, and then answering some more questions. Part 3 will last approximately 60 minutes. If you do not intend to participate in Part 3 of the study, please do not complete Part 2.

Keywords: survey, politics, ideology, opinions

***Zoom portion mTurk post (only seen by scheduled participants)***

Project Name: Ideological Conversations Study (Part 3)

Title: Complete interactive task with others who have similar or different ideological views

Description: You will answer a few questions, interact (via video chat) with others who share similar and different ideological views from you, and then answer some more questions. The experiment will last approximately 60 minutes.

Keywords: survey, politics, science, opinions

**PRESCREENER QUESTIONS**---------------------------------------------------------------------------------------------------

**Q1.** How much do you agree with the following statement:

“The father of an unintended pregnancy **should** have the right to be involved in the decision-making process for an abortion.”

Strongly Somewhat Neither agree Slightly Strongly

disagree Disagree disagree nor disagree agree Agree agree

1 2 3 4 5 6 7


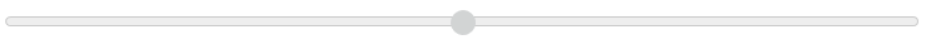


**Q2.** Imagine you are having a conversation with a stranger who disagrees with you on the issue of whether the father of an unwanted pregnancy should have the right to be involved during the decision-making process for an abortion. How likely is it that you would **defend your own perspective even if it generates conflict:**

Extremely Moderately Extremely

unlikely likely likely

1 2 3 4 5 6 7


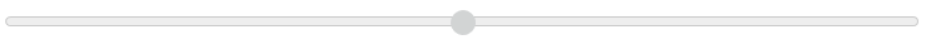


**Q3.** How much do you agree with the following statement:

“We **should not** force people into the categories of male or female, gender is a spectrum.”

Strongly Somewhat Neither agree Slightly Strongly

disagree Disagree disagree nor disagree agree Agree agree

1 2 3 4 5 6 7


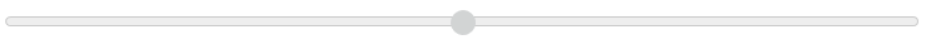


**Q4.** Imagine you are having a conversation with a stranger who disagrees with you on the issue of whether gender is a spectrum. How likely is it that you would **defend your own perspective even if it generates conflict:**

Extremely Moderately Extremely

unlikely likely likely

1 2 3 4 5 6 7


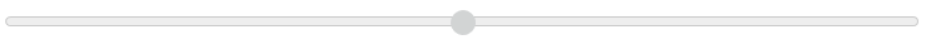


**Q5.** How much do you agree with the following statement:

“Cities **should** defund the police to combat systemic discrimination.”

Strongly Somewhat Neither agree Slightly Strongly

disagree Disagree disagree nor disagree agree Agree agree

1 2 3 4 5 6 7


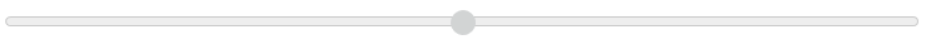


**Q6.** Imagine you are having a conversation with a stranger who disagrees with you on the issue of whether cities should defund the police to combat systemic discrimination. How likely is it that you would **defend your own perspective even if it generates conflict:**

Extremely Moderately Extremely

unlikely likely likely

1 2 3 4 5 6 7


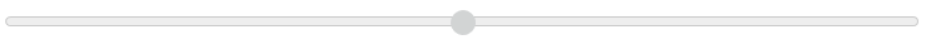


**Q7.** How much do you agree with the following statement:

“Colleges **should** use affirmative action policies, such as considering an applicant's ethnicity, income level, etc., when deciding admissions.”

Strongly Somewhat Neither agree Slightly Strongly

disagree Disagree disagree nor disagree agree Agree agree

1 2 3 4 5 6 7


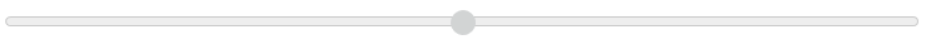


**Q8.** Imagine you are having a conversation with a stranger who disagrees with you on the issue of whether colleges should use affirmative action. How likely is it that you would **defend your own perspective even if it generates conflict:**

Extremely Moderately Extremely

unlikely likely likely

1 2 3 4 5 6 7


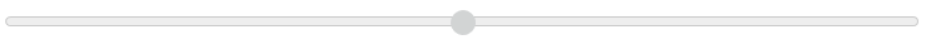


**Q9.** How much do you agree with the following statement:

"Please select Agree (6) as the answer to this question to demonstrate you are not a bot."

Strongly Somewhat Neither agree Slightly Strongly

disagree Disagree disagree nor disagree agree Agree agree

1 2 3 4 5 6 7


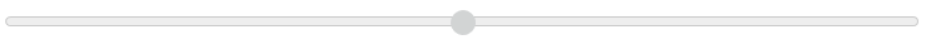


**Q10.** How much do you agree with the following statement:

“Private businesses **should** have the right to refuse service on the basis of religious exemptions.”

Strongly Somewhat Neither agree Slightly Strongly

disagree Disagree disagree nor disagree agree Agree agree

1 2 3 4 5 6 7


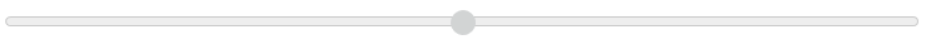


**Q11.** Imagine you are having a conversation with a stranger who disagrees with you on the issue of whether businesses have the right to refuse service based on religious exemptions. How likely is it that you would **defend your own perspective even if it generates conflict:**

Extremely Moderately Extremely

unlikely likely likely

1 2 3 4 5 6 7


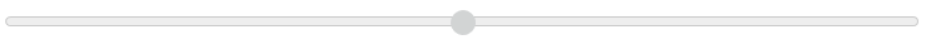


**PRE-STUDY: *ATTITUDE MEASURES***----------------------------------------------------------------------------------------

**Q1.** Please read the statement carefully, then indicate how strongly you agree with the issue as it is stated below:

"Colleges **should use** affirmative action policies, such as considering an applicant's ethnicity, income level, etc., when deciding admissions."

Strongly Somewhat Neither agree Slightly Strongly

disagree Disagree disagree nor disagree agree Agree agree

1 2 3 4 5 6 7


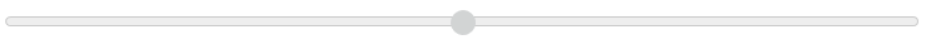


**Q2.** How much do you care about the issue of whether colleges should use affirmative action?

Not at all Somewhat Moderately Extremely

|  |  |  |  |  |
| --- | --- | --- | --- | --- |

**Q3.** How informed do you feel about the issue of whether colleges should use affirmative action?

Not at all Somewhat Moderately Extremely

|  |  |  |  |  |
| --- | --- | --- | --- | --- |

**Q4.** How important is the issue of whether colleges should use affirmative action?

Not at all Somewhat Moderately Extremely

|  |  |  |  |  |
| --- | --- | --- | --- | --- |

**Q5.** How much more correct is your opinion on this issue compared to the opposite opinion?

o No more correct than the other opinion

o Somewhat more correct than the other opinion

o Much more correct than the other opinion

o Totally correct--mine is the only correct opinion

**Q6.** What percentage of **the public** do you think share the same view as you on this issue?

None All

0 10 20 30 40 50 60 70 80 90 100

(Percentage)


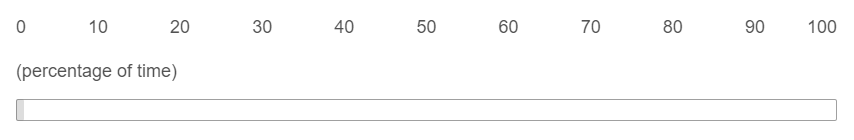


**Q7.** How much do you think **the average person** agrees with this statement?

"Colleges **should use** affirmative action policies, such as considering an applicant's ethnicity, income level, etc., when deciding admissions."

Select one:

Strongly Somewhat Neither agree Slightly Strongly

disagree Disagree disagree nor disagree agree Agree agree

|  |  |  |  |  |  |  |
| --- | --- | --- | --- | --- | --- | --- |

**Q8.** "Colleges **should use** affirmative action policies, such as considering an applicant's ethnicity, income level, etc., when deciding admissions."

Below you will **rate how you feel,** favorably or unfavorably, **towards people with specific opinions on the above statement**:

Extremely No feeling Extremely

unfavorable at all favorable

1 2 3 4 5 6 7

I feel ______ towards **people who STRONGLY AGREE** with the statement.


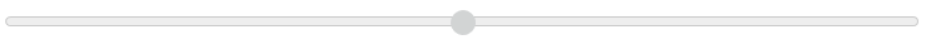


I feel ______ towards **people who STRONGLY AGREE** with the statement.


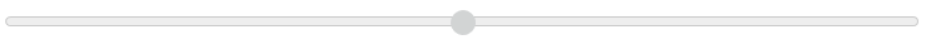


**Q9. What positions** could **other people hold** on this issue that would **NOT** **bother or annoy** you?

Please use the sliders to mark the **boundaries** of the range of opinions that do not bother or annoy you.

"Colleges **should use** affirmative action policies, such as considering an applicant's ethnicity, income level, etc., when deciding admissions."

The sliders will start at your own position. You should move them so that they mark the **farthest** positions to the left and right from your own opinion that do not bother or annoy you.

Strongly Somewhat Neither agree Slightly Strongly

disagree Disagree disagree nor disagree agree Agree agree

1 2 3 4 5 6 7

Select the farthest position to the left of your opinion that does not bother or annoy you

<<<<


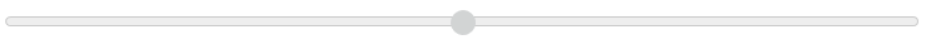


Select the farthest position to the right of your opinion that does not bother or annoy you

>>>>


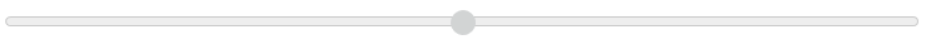


**Q10.** In a future experiment, you may interact with someone who has the opposite opinion as you on the following issue:

“Colleges **should use** affirmative action policies, such as considering an applicant's ethnicity, income level, etc., when deciding admissions.”

What position do you think this person would have on this issue?

Strongly Somewhat Neither agree Slightly Strongly

disagree Disagree disagree nor disagree agree Agree agree

1 2 3 4 5 6 7


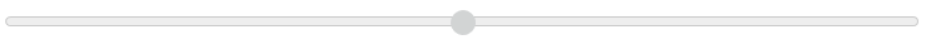


**Q11.** In a later part of this experiment, you may have a conversation with someone who has the opposite opinion as you on the following issue:

"Colleges **should use** affirmative action policies, such as considering an applicant's ethnicity, income level, etc., when deciding admissions."

Please answer the following questions concerning how you feel about having a conversation with someone who **disagrees** with you on this issue.

|  | Strongly disagree | Disagree | Somewhat disagree | Neither agree nor disagree | Slightly agree | Agree | Strongly agree |
| --- | --- | --- | --- | --- | --- | --- | --- |
| I feel this interaction would be enjoyable |  |  |  |  |  |  |  |
| I feel this interaction would be very stressful |  |  |  |  |  |  |  |
| I would be afraid of being judged by the others present |  |  |  |  |  |  |  |
| I would feel comfortable in this interaction |  |  |  |  |  |  |  |
| I feel the interaction would be difficult to get through |  |  |  |  |  |  |  |
| I believe their ideas would be valid |  |  |  |  |  |  |  |
| I believe their ideas would be convincing |  |  |  |  |  |  |  |
| I think I could respect their opinions |  |  |  |  |  |  |  |
| I believe their statements would be driven by emotion |  |  |  |  |  |  |  |
| I believe their statements would be driven by logic |  |  |  |  |  |  |  |
| I think I would like them as a person |  |  |  |  |  |  |  |

**Q12.** What percentage of the time would predict that you would be **getting along** with the person of the opposite opinion during this conversation?

0 10 20 30 40 50 60 70 80 90 100

(Percentage of time)


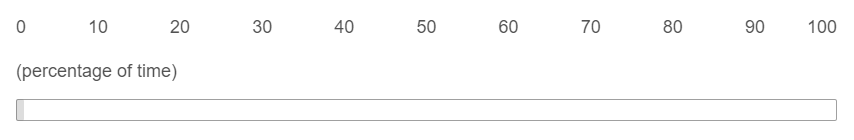


**Q13.** What percentage of the time would you predict that you would be **in conflict** with the person of the opposite opinion during this conversation?

0 10 20 30 40 50 60 70 80 90 100

(Percentage of time)


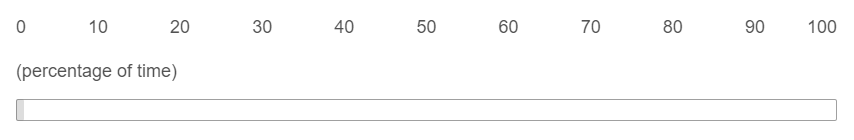


**POST-STUDY: *ATTITUDE MEASURES***--------------------------------------------------------------------------------------

We are going to ask you some of the same questions from the survey you took before the conversation portion of the study. Your responses should reflect how you feel at this moment. Please be as honest as possible.

**Q1.** Please indicate how strongly you agree with the following statement:

"Colleges **should** use affirmative action policies, such as considering an applicant's ethnicity, income level, etc., when deciding admissions."

Strongly Somewhat Neither agree Slightly Strongly

disagree Disagree disagree nor disagree agree Agree agree

1 2 3 4 5 6 7


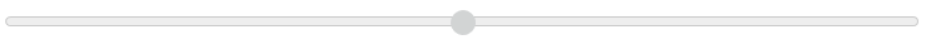


**Q2.** How much do you care about the issue of whether colleges should use affirmative action?

Not at all Somewhat Moderately Extremely

|  |  |  |  |  |
| --- | --- | --- | --- | --- |

**Q3.** How informed do you feel about the issue of whether colleges should use affirmative action?

Not at all Somewhat Moderately Extremely

|  |  |  |  |  |
| --- | --- | --- | --- | --- |

**Q4.** How important is the issue of whether colleges should use affirmative action?

Not at all Somewhat Moderately Extremely

|  |  |  |  |  |
| --- | --- | --- | --- | --- |

**Q5.** How much more correct is your opinion on this issue compared to the opposite opinion?

o No more correct than the other opinion

o Somewhat more correct than the other opinion

o Much more correct than the other opinion

o Totally correct--mine is the only correct opinion

**Q6.** What percentage of **the public** do you think share the same view as you on this issue?

None All

0 10 20 30 40 50 60 70 80 90 100

(Percentage)


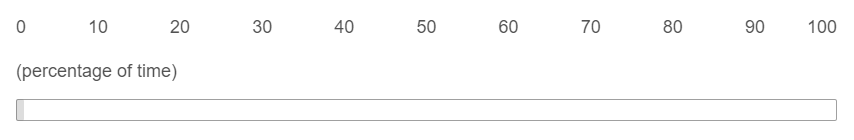


**Q7.** How much do you think **the average person** agrees with this statement?

"Colleges **should use** affirmative action policies, such as considering an applicant's ethnicity, income level, etc., when deciding admissions."

Select one:

Strongly Somewhat Neither agree Slightly Strongly

disagree Disagree disagree nor disagree agree Agree agree

|  |  |  |  |  |  |  |
| --- | --- | --- | --- | --- | --- | --- |

**Q8.** "Colleges **should use** affirmative action policies, such as considering an applicant's ethnicity, income level, etc., when deciding admissions."

Below you will **rate how you feel,** favorably or unfavorably, **towards people with specific opinions on the above statement**:

Extremely No feeling Extremely

unfavorable at all favorable

1 2 3 4 5 6 7

I feel ______ towards **people who STRONGLY AGREE** with the statement.


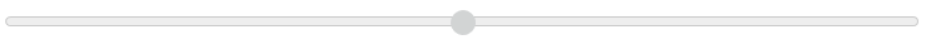


I feel ______ towards **people who STRONGLY AGREE** with the statement.


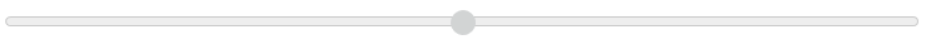


**Q10. What positions** could **other people hold** on this issue that would **NOT** **bother or annoy** you?

Please use the sliders to mark the **boundaries** of the range of opinions that do not bother or annoy you.

"Colleges **should use** affirmative action policies, such as considering an applicant's ethnicity, income level, etc., when deciding admissions."

The sliders will start at your own position. You should move them so that they mark the **farthest** positions to the left and right from your own opinion that do not bother or annoy you.

Strongly Somewhat Neither agree Slightly Strongly

disagree Disagree disagree nor disagree agree Agree agree

1 2 3 4 5 6 7

Select the farthest position to the left of your opinion that does not bother or annoy you

<<<<


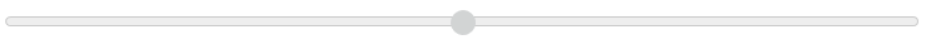


Select the farthest position to the right of your opinion that does not bother or annoy you

>>>>


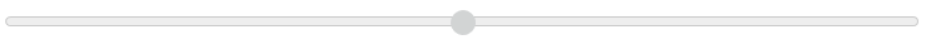


**Q9.** How much would you say the **first conversation** had an effect on your opinion about the issue of whether colleges should use affirmative action policies?

No effect Some effect Moderate effect Large effect

|  |  |  |  |  |
| --- | --- | --- | --- | --- |

**Q10.** How much would you say the **second conversation** had an effect on your opinion about the issue of whether colleges should use affirmative action policies?

No effect Some effect Moderate effect Large effect

|  |  |  |  |  |
| --- | --- | --- | --- | --- |

**Q11.** You replied that one or two of the conversations had an effect on your opinion of the issue. Did the conversation weaken or strengthen your original opinion?

Greatly Moderately Somewhat Somewhat Moderately Greatly

weakened weakened weakened No effect strengthened strengthened strengthened

1 2 3 4 5 6 7

First conversation


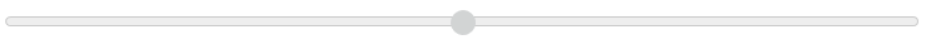


Second conversation


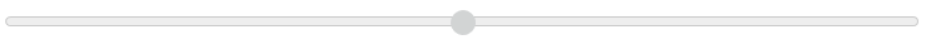


**Q12.** (Optional) What do you think is the most important aspect of the issue regarding whether colleges should use affirmative action policies?

__________________________________________________________________________________________________________________________________________________________________________________________

***Note.*** *These questions were asked for the first conversation and same/similar attitude partner but have been removed for brevity*

**Q13**. Consider the person you talked to during the second conversation, who had an opposing opinion about the statement.

How much do you think this person agrees with this statement?

"Colleges **should** use affirmative action policies, such as considering an applicant's ethnicity, income level, etc., when deciding admissions."

Strongly Somewhat Neither agree Slightly Strongly

disagree Disagree disagree nor disagree agree Agree agree

1 2 3 4 5 6 7


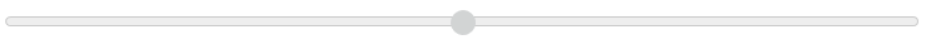


**Q14.** The following questions will be about the person who held the **opposite** opinion from the **second** conversation. Indicate how much you agree with each statement below. Please be honest with your answers.

|  | Strongly disagree | Disagree | Somewhat disagree | Neither agree nor disagree | Slightly agree | Agree | Strongly agree |
| --- | --- | --- | --- | --- | --- | --- | --- |
| I liked them as a person |  |  |  |  |  |  |  |
| We are very different types of people |  |  |  |  |  |  |  |
| I think they would agree with me on other important issues |  |  |  |  |  |  |  |
| I wouldn’t mind talking to them again |  |  |  |  |  |  |  |

**Q15.** The following questions will be about how you perceived the interaction between yourself and the person who held the **opposite** opinion in the **second** conversation.

|  | Strongly disagree | Disagree | Somewhat disagree | Neither agree nor disagree | Slightly agree | Agree | Strongly agree |
| --- | --- | --- | --- | --- | --- | --- | --- |
| I was motivated to engage in the discussion |  |  |  |  |  |  |  |
| They were motivated to engage in the discussion |  |  |  |  |  |  |  |
| I felt the interaction was difficult to get through |  |  |  |  |  |  |  |
| We handled differences of opinions by addressing them directly |  |  |  |  |  |  |  |
| I feel this interaction was very stressful |  |  |  |  |  |  |  |
| I felt comfortable in the interaction |  |  |  |  |  |  |  |
| I felt the interaction was enjoyable |  |  |  |  |  |  |  |
| They dominated the conversation |  |  |  |  |  |  |  |
| I dominated the conversation |  |  |  |  |  |  |  |
| They listened carefully to me |  |  |  |  |  |  |  |
| I listened carefully to them |  |  |  |  |  |  |  |
| I felt judged by this person |  |  |  |  |  |  |  |
| I felt like I was able to say what I wanted |  |  |  |  |  |  |  |
| I felt like they were able to say what they wanted |  |  |  |  |  |  |  |

**Q16.** The following questions will be about the content of the interaction between you and the person who held the **opposite** opinion in the **second** conversation.

|  | Strongly disagree | Disagree | Somewhat disagree | Neither agree nor disagree | Slightly agree | Agree | Strongly agree |
| --- | --- | --- | --- | --- | --- | --- | --- |
| I thought their ideas were valid |  |  |  |  |  |  |  |
| I thought their ideas were convincing |  |  |  |  |  |  |  |
| I could respect their opinions |  |  |  |  |  |  |  |
| We had very different background information about the topic |  |  |  |  |  |  |  |
| Their statements were driven by emotion |  |  |  |  |  |  |  |
| Their statements were driven by logic |  |  |  |  |  |  |  |
| My statements were driven by emotion |  |  |  |  |  |  |  |
| My statements were driven by logic |  |  |  |  |  |  |  |

**Q17.** What percentage of the time would you say that you two were **getting along**?

0 10 20 30 40 50 60 70 80 90 100

(Percentage of time)


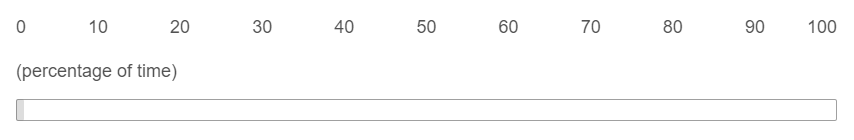


**Q18.** What percentage of the time would you say that you two were **in conflict**?

0 10 20 30 40 50 60 70 80 90 100

(Percentage of time)


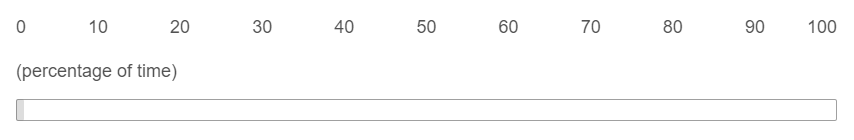


**Q19.** From the second conversation, what do you think the person's general ideological views are?

Strongly Somewhat Slightly Strongly

Conservative Conservative conservative Moderate liberal Liberal liberal

|  |  |  |  |  |  |  |
| --- | --- | --- | --- | --- | --- | --- |

**Q20.** How important do you think these views are to them?

Not at all Somewhat Moderately Extremely

|  |  |  |  |  |
| --- | --- | --- | --- | --- |

**Q21.** To what extent do you think their ideological views are similar to your own?

Not at all Somewhat Moderately Extremely

|  |  |  |  |  |
| --- | --- | --- | --- | --- |

**Q22.** Do you believe having/if the **person from the first** conversation sit in on your **second** conversation made it easier to converse and be honest about your opinion in the **second** conversation?

Definitely Probably not Unsure Probably yes Definitely yes

**Q23.** Do you believe having/if the **person from the first** conversation sit in on your **second** conversation put more pressure on you to defend your opinion against the person in the **second** conversation?

Definitely Probably not Unsure Probably yes Definitely yes

**Q26.** This is the last question on the survey. Please use the box below to share any additional comments (optional).

_______________________________________________________________________________________________________________________________________________________________________________________________________________________________________________________________________________________

**CODER QUESTIONS**---------------------------------------------------------------------------------------------------

***Note.*** *Coders were first asked about each conversation partner separately (Q’s 1-11), then asked about the conversation as a whole (Q’s 12-15).*

***Single participant questions***

Instructions: The following set of questions will be about the **participant with ID starting with a 1**. Please make sure you are answering all the questions on this page about the participant with ID starting with a 1 and not the other participant.

What is the participants unique 3 digit IPC code **(STARTS WITH A 1)**? This can be found on the conversation video you just watched (e.g., 102, 124).

Please doublecheck that you are rating the participant who has ID starting with 1

**Q1.** The following questions will be about how the participant with ID starting with a __ viewed their conversation partner. Indicate how much you agree with each statement below.

|  | Strongly disagree | Disagree | Somewhat disagree | Neither agree nor disagree | Slightly agree | Agree | Strongly agree |
| --- | --- | --- | --- | --- | --- | --- | --- |
| They liked them as a person |  |  |  |  |  |  |  |
| They could respect their partners opinions |  |  |  |  |  |  |  |
| They looked convinced by their partners statements |  |  |  |  |  |  |  |

**Q2.** The following questions will be about how the participant with ID starting with a __ viewed their conversation partner. Indicate how much you agree with each statement below.

The following questions will be about your perception of the participant with ID starting with a __ in the interaction with their conversation partner.

|  | Strongly disagree | Disagree | Somewhat disagree | Neither agree nor disagree | Slightly agree | Agree | Strongly agree |
| --- | --- | --- | --- | --- | --- | --- | --- |
| They were motivated to engage in the discussion |  |  |  |  |  |  |  |
| The interaction looked difficult to get through for them |  |  |  |  |  |  |  |
| They handled differences of opinions by addressing them directly |  |  |  |  |  |  |  |
| The interaction looked very stressful for them |  |  |  |  |  |  |  |
| They looked comfortable in the interaction |  |  |  |  |  |  |  |
| They looked nervous in the interaction |  |  |  |  |  |  |  |
| They looked frustrated in the interaction |  |  |  |  |  |  |  |
| It looked like they enjoyed the interaction |  |  |  |  |  |  |  |
| They dominated the conversation |  |  |  |  |  |  |  |
| They listened carefully |  |  |  |  |  |  |  |
| They looked like they were able to say what they wanted |  |  |  |  |  |  |  |
| They hesitated to express disagreement |  |  |  |  |  |  |  |
| They heatedly disagreed |  |  |  |  |  |  |  |
| They instigated conflict |  |  |  |  |  |  |  |
| They used an inflammatory tone |  |  |  |  |  |  |  |
| They seemed to be arguing in bad faith |  |  |  |  |  |  |  |

**Q3.** What percentage of the time would you say that the participant with ID starting with a __ was **getting along** with their partner of the opposite opinion?

0 10 20 30 40 50 60 70 80 90 100

(Percentage of time)


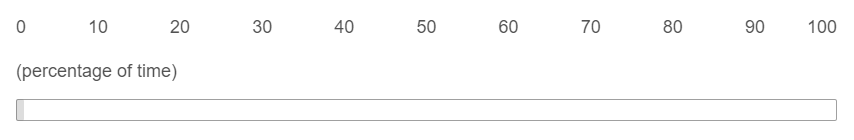


**Q4.** What percentage of the time would you say the participant with ID starting with a __ was in **conflict** with their partner of the opposite opinion?

0 10 20 30 40 50 60 70 80 90 100

(Percentage of time)


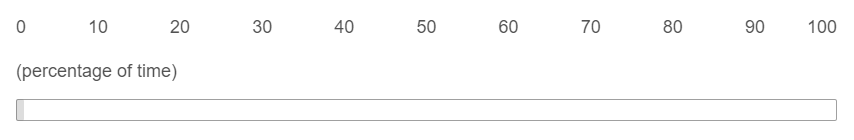


**Q5.** What do you believe the participant with ID starting with a __ attitude was at the **START** of the conversation for the issue as stated:

“Colleges **should use** affirmative action policies, such as considering an applicant's ethnicity, income level, etc., when deciding admissions.”

Strongly Somewhat Neither agree Slightly Strongly

disagree Disagree disagree nor disagree agree Agree agree

1 2 3 4 5 6 7


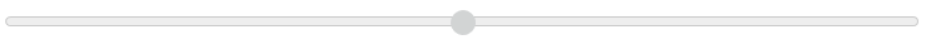


**Q6.** How much would you say the conversation had an effect on the participant with ID starting with a __ opinion about the issue?

No effect Some effect Moderate effect Large effect

|  |  |  |  |  |
| --- | --- | --- | --- | --- |

**Q7.** Do you think the conversation **weakened** or **strengthened** the participant with ID starting with a __ original opinion? (If no effect click "No effect")

Greatly Moderately Somewhat Somewhat Moderately Greatly

weakened weakened weakened No effect strengthened strengthened strengthened

1 2 3 4 5 6 7

Direction of effect


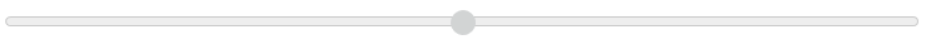


**Q8.** What do you believe the participant with ID starting with a __ attitude was at the **END** of the conversation for the issue as stated:

“Colleges **should use** affirmative action policies, such as considering an applicant's ethnicity, income level, etc., when deciding admissions.”

Strongly Somewhat Neither agree Slightly Strongly

disagree Disagree disagree nor disagree agree Agree agree

1 2 3 4 5 6 7


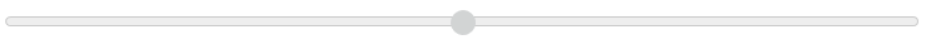


**Q9.** How would you describe the participant with ID starting with a __ political views?

Strongly Somewhat Slightly Strongly

conservative Conservative conservative Moderate liberal Liberal liberal

|  |  |  |  |  |  |  |
| --- | --- | --- | --- | --- | --- | --- |

**Q10.** After the conversation, how much more correct do you think the participant with ID starting with a ___ believes their opinion on this issue is compared to the opposite opinion?

o No more correct than the other opinion

o Somewhat more correct than the other opinion

o Much more correct than the other opinion

o Totally correct--theirs is the only correct opinion

**Q11.** How much do you feel that the participant with ID starting with a __ was:

|  | None | Seldom | Sometimes | Often | Always |
| --- | --- | --- | --- | --- | --- |
| Privately disagreeing in their head, but not outwardly showing it? |  |  |  |  |  |
| Outwardly expressing their disagreement? |  |  |  |  |  |

***Dyad based questions***

**Q12.** How much do you agree that the two participants **seemed like very different types of people**?

Strongly Somewhat Neither agree Slightly Strongly

disagree Disagree disagree nor disagree agree Agree agree

|  |  |  |  |  |  |  |
| --- | --- | --- | --- | --- | --- | --- |

**Q13.** How would you describe the conversation in terms of how much participants agreed with each other?

o Disagreed on everything

o Moderately more disagreement

o Somewhat more disagreement

o Neither agreed nor disagreed

o Somewhat more agreement

o Moderately more agreement

o Agreed on everything

**Q14.** How would you describe the conversation in terms of the type of conflict in the conversation, if any?

o No conflict

o Extremely calm

o Calm

o Somewhat calm

o Conflict but neither calm nor heated

o Somewhat heated

o Heated

o Extremely heated

**Q15.** Overall, how much **hidden** and **outward** conflict was there in this conversation?

|  | None | Seldom | Sometimes | Often | Constant/Always |
| --- | --- | --- | --- | --- | --- |
| Hidden conflict |  |  |  |  |  |
| Outward conflict |  |  |  |  |  |
